# Functional mapping of brain synapses by the enriching activity-marker SynaptoZip

**DOI:** 10.1038/s41467-017-01335-4

**Published:** 2017-10-31

**Authors:** Mattia Ferro, Jacopo Lamanna, Maddalena Ripamonti, Gabriella Racchetti, Alessandro Arena, Sara Spadini, Giovanni Montesano, Riccardo Cortese, Vincenzo Zimarino, Antonio Malgaroli

**Affiliations:** 1 Università Vita-Salute San Raffaele, Milan, 20132 Italy; 2 Neurobiology of Learning Unit, Division of Neuroscience, Scientific Institute Ospedale San Raffaele, Milan, 20132 Italy; 3 Psychiatry and Clinical Psychobiology Unit, Division of Neuroscience, Scientific Institute Ospedale San Raffaele, Milan, 20132 Italy; 4 Keires AG, Basel, CH 4051 Switzerland; 5 Present Address: Department of Physiology, Institute of Basal Medical Sciences, University of Oslo, Oslo, 0315 Norway; 6 Present Address: Dipartimento Testa-Collo, San Paolo Hospital, University of Milan, Milan, 20122 Italy

## Abstract

Ideally, elucidating the role of specific brain circuits in animal behavior would require the ability to measure activity at all involved synapses, possibly with unrestricted field of view, thus even at those boutons deeply located into the brain. Here, we introduce and validate an efficient scheme reporting synaptic vesicle cycling in vivo. This is based on SynaptoZip, a genetically encoded molecule deploying in the vesicular lumen a bait moiety designed to capture upon exocytosis a labeled alien peptide, Synbond. The resulting signal is cumulative and stores the number of cycling events occurring at individual synapses. Since this functional signal is enduring and measurable both online and ex post, SynaptoZip provides a unique method for the analysis of the history of synaptic activity in regions several millimeters below the brain surface. We show its broad applicability by reporting stimulus-evoked and spontaneous circuit activity in wide cortical fields, in anesthetized and freely moving animals.

## Abstract (web summary)

Visualization of synaptic activity in the living brain is challenging. This study devises a simple and efficient scheme that reports synaptic vesicle recycling in vivo using SynaptoZip, a genetically encoded sensor of past synaptic activities.

## Introduction

Recent years have witnessed the birth of a variety of molecular tools to control or sense neuronal activity (see for review^1–4^). The latter family includes several genetically encoded fluorescent indicators that can detect changes in either membrane voltage or calcium concentration (see for review^5–9^). Ideally, a complete understanding of the physiological role of a specific area or circuit would require the ability to map synaptic transmission independently from the electrical activity of neurons. Following the introduction of FM dyes in the 90s^10^, there have been significant advances in the development of methods that can probe synaptic communication^11–17^, which include elegant genetically encoded fluorescent indicators of vesicle exo-endocytosis^11,12,14,16,18^. The most important inherent constraint of these techniques is their limited applicability to the analysis of the living mammalian brain. This is due to the transient nature of generated signals, whose detection requires online optical imaging. Besides the requirement of expensive and complex instrumentations, it can only access, with the necessary optical resolution, synapses that are no more than a millimeter below the brain surface, because of reduced light penetration in tissue^19^. This warrants the development of alternative approaches to monitor changes in synaptic activity that could be applied to brain circuits. Here, we have expanded the repertoire of available synaptic methods to include a reporter that, due to the binding of a peptide ligand^20^, generates a very specific and stable coiled–coil interaction^21^. The output relates to presynaptic bouton activity and is a long-lasting integration signal reporting the history of vesicle cycling, hence well-suited for both online and retrospective circuit analysis. We provide proof of principle of this strategy and show here its effectiveness in evaluating activity changes at the level of individual brain synapses, following in vivo experimental interventions.

## Results

### Design and validation of SynaptoZip (SZ)

**Fig. 1:**
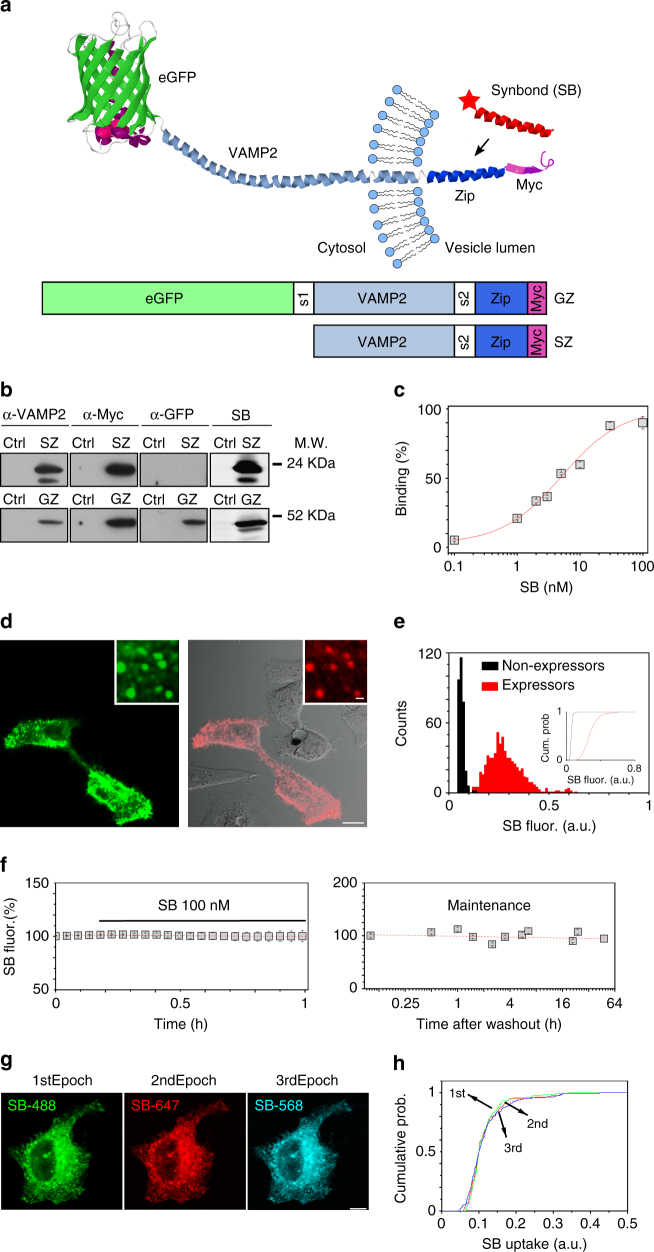
Design and characterization of SynaptoZip. **a** Cartoon depicting the reporter system; box diagrams illustrate SZ moieties. S1, S2: spacer sequences; Zip: Acid-p1. **b** WB of transfected (GZ, SZ) and non-transfected (Ctrl) Hela cells, detection with anti-VAMP2, anti-Myc epitope tag, anti-GFP antibodies, and with SB-Alexa647 (see also Supplementary Fig. 1a). **c** Analysis of Zip-Bond binding in live Hela cells (the red line is the logistic fitting, adj. *R* ^2^ = 0.98, *K*_d_ = 5.09 nM; *N*_0.1nM_ = 38, *N*_1nM_ = 65, *N*_2nM_ = 45, *N*_3nM_ = 80, *N*_5nM_ = 59, *N*_10nM_ = 39, *N*_30nM_ = 48, *N*_100nM_ = 36; mean ± s.e.m.). **d** SB uptake in a mixture of transfected and non-transfected Hela cells (green, eGFP; red, SB and DIC image; SB 5 nM, 1 h incubation). **e** Histograms of SB fluorescence and their cumulative representation (Inset; *N* = 320, non-expressing cells; *N* = 636, GZ-expressing cells; SB_expr_ = 0.284 ± 0.089, SB_non-expr_ = 0.063 ± 0.014, mean ± SD; *p* < 0.01, KS test). **f** Analysis of the fate of internalized SB in SZ-expressing Hela cells. Left, internalized SB-Alexa647 was not displaced by excess of SB-Alexa488 (time-lapse imaging, *N* = 6; mean ± s.e.m.). Right, vesicular SB remains up to 48 h from extracellular washout (*N*_5min_ = 71, *N*_0.5h_ = 60, *N*_1h_ = 47, *N*_1.5h_ = 60, *N*_2.5h_ = 53, *N*_3.5h_ = 73, *N*_5.5h_ = 42, *N*_6.5h_ = 39, *N*_21h_ = 37, *N*_24h_ = 50, *N*_48h_ = 47; mean ± s.e.m.). **g** Hela cells expressing non-fluorescent SZ were incubated sequentially with SB-Alexa488, SB-Alexa647, SB-Alexa568. **h** Cumulative histograms of cell fluorescence from *N* = 92 cells as in **g**, illustrating the labeling similarity among the epochs (*p* ≥ 0.26; KS test). Scale bars, 10 μm, 1 μm inset (**d**) and 10 μm (**g**)

To develop SynaptoZip, one component of the Velcro coiled-coil heterodimer^20^, the Zip module, was fused to the intraluminal C-terminus of VAMP-2/Synaptobrevin2, followed by a Myc tag. Its fluorescent variant, eGFP-SynaptoZip (GZ), was generated by adding eGFP to the cytosolic N-terminus (Fig. 1a). The other component of Velcro was chemically synthetized for use as soluble SynaptoZip binder, Synbond (SB; 3.9 KDa; Fig. 1a). Both SynaptoZip variants (GZ and SZ) expressed in Hela cells were found to be localized in the endoplasmic reticulum, in the Golgi complex, and in a population of highly motile vesicles, presumably endocytic vesicles/recycling endosomes, due to their colocalization with internalized transferrin (data not shown; see below). Immunoblotting of Hela cells expressing SynaptoZip revealed a major band of 28 KDa for SZ and 55 KDa for GZ, which were recognized by anti-VAMP2, as well as by anti-Myc antibodies (Fig. 1b; Supplementary Fig. 1a). Interestingly, fluorescent SB directly labeled the appropriate M.W. bands on blot membranes, and consistent results were obtained with neuronal extracts (Fig. 1b; Supplementary Fig. 1a, b). To test whether extracellular SB molecules were competent for SynaptoZip binding and uptake during cycles of constitutive vesicular recycling, SynaptoZip-expressing Hela cells were incubated with fluorescent SB molecules (5 nM; SB-Alexa647), for periods ranging from 1 to 60 min (Supplementary Fig. 2a, b). SB concentration was selected based on in vitro binding affinity^20^ and live binding experiments on Hela cells (Fig. 1c; *K*_d_ = 5.09 nM; *N* = 36–80; logistic fitting, adj. *R* ^2^ = 0.98). As shown in Fig. 1d, in a mixture of expressing and non-expressing cells, SB was selectively internalized in expressors, following ongoing or constitutive vesicular exo-endocytosis (Fig. 1d; GZ left; SB and DIC image, right).

Since the pool of SB binding sites in Hela cells should be fairly stable (within a short-time window), we evaluated the behavior of SB uptake over time (Supplementary Fig. 2a, b). Specific SB internalization in GZ-expressing Hela cells could be detected already at 1–2 min from its application (1 min: *N*_expr_ = 64, SB_expr_ = 0.018 ± 0.017, *N*_non-expr_ = 49, SB_non-expr_ = 10^−4^ ± 0.0022; mean ± SD; *p* < 10^−4^ Wilcoxon rank sum test), and at later time points its intracellular accumulation increased approaching a plateau level (Supplementary Fig. 2a, b), presumably because of the progressive saturation of the cycling sites by elevated constitutive exo-endocytosis. When cells were magnified, the red SB labeling could be fully attributed to eGFP-fluorescent endocytic vesicles expressing SB binding sites (Fig. 1d, insets). Consistent results were obtained with the eGFP lacking variant SZ (see below). SB-labeled vesicles showed physiological dynamics characteristic of endocytic vesicles/recycling endosomes (Supplementary Movie 1). SB uptake was not seen at 4 °C (data not shown), suggesting the requirement for constitutive exo-endocytosis^22^ in SB internalization. Chemical fixation (10–15 min, 4 °C, 4% paraformaldehyde, PFA) did not alter SB retention and a comparable estimate of the amount of internalized SB could be obtained before and after fixation (data not shown).

As shown by the histograms (Fig. 1e), SB uptake occurred essentially by a specific interaction of SB with the luminal epitope of GZ, and not by unspecific fluid phase endocytosis, because only GZ-positive cells showed a strong and fast SB uptake signal (Fig. 1d, e; SB 5 nM, 60 min incubation; *N* = 320, non-expressing cells; *N* = 636, GZ-expressing cells; SB_expr_ = 0.284 ± 0.089, SB_non-expr_ = 0.063 ± 0.014, mean ± SD; *p* < 0.01, Kolmogorov–Smirnov test (KS)). Co-application of SB and antibodies against the intraluminal Myc tag resulted in the co-localization of both molecules in the same GZ-positive vesicles (data not shown). After long-term exposure to SB, its uptake linearly correlated with GZ expression level, both assayed in the sub-membrane region of Hela cells (Supplementary Fig. 2c), where constitutive endocytic vesicle recycling takes place. Similar results were obtained by analyzing individual vesicles (Supplementary Fig. 2d, e). Altogether these results confirm that SynaptoZip is correctly inserted and oriented in the vesicle membrane. Exocytosis exposes to the extracellular environment the C-terminal Zip module which then binds extracellular SB, leading to its very specific entrapment in the endocytic vesicle.

Despite continuous constitutive vesicle recycling, pre-bound SB-Alexa647 (5 nM, O/N incubation, 37 °C) was neither significantly displaced by subsequent application of an excess of SB-Alexa488 (Fig. 1f, left panel; time lapse imaging; SB-Alexa488, 100 nM, 37 °C, *N* = 6 cells; *p* = 0.84, Wilcoxon signed-rank test; analyzed windows 0–10 vs. 50–60 min), nor spontaneously lost up to 48 h after its vesicular uptake (Fig. 1f, right panel; *N* = 38–74 cells per data point; Pearson correlation: *ρ* = −0.047, *p* = 0.26). This suggests that vesicular SB is not free but it is bound to GZ in a stable complex, as predicted by Velcro in vitro stability^20^. Sequential brief incubations (10 min, 37 °C) with three different SB fluorescent variants (SB-Alexa488, SB-Alexa568, and SB-Alexa647, 5 nM each) resulted in comparable macroscopic patterns of vesicular staining (Fig. 1g). When cumulative fluorescence distributions from the three sequential epochs were plotted, these nearly coincided (Fig. 1h; average fluorescence; *N* = 92 cells; *p* ≥ 0.26; KS test). In the above experimental conditions, the absence of a significant distortion of the SB uptake distribution, over time and following repeated incubations, indicates good assay reproducibility, without saturation of available SB-binding sites. As a whole, these results show that the amount of the SZ–SB complex can effectively and quantitatively report vesicular exo-endocytosis in a cultured cell line, generating a persistent and reliable functional signal.

### SynaptoZip reports synaptic activity

To test whether this approach could be applied to monitor synaptic exo-endocytosis, we expressed GZ in cultured hippocampal neurons and in various brain regions (hippocampus, visual thalamus, primary visual, and prefrontal cortices). In all preparations investigated, clear eGFP fluorescent presynaptic varicosities could be seen. These were confirmed as bona fide synapses, because of the large extent of colocalization with pre-synaptic and post-synaptic markers (Supplementary Fig. 3a, b).

**Fig. 2:**
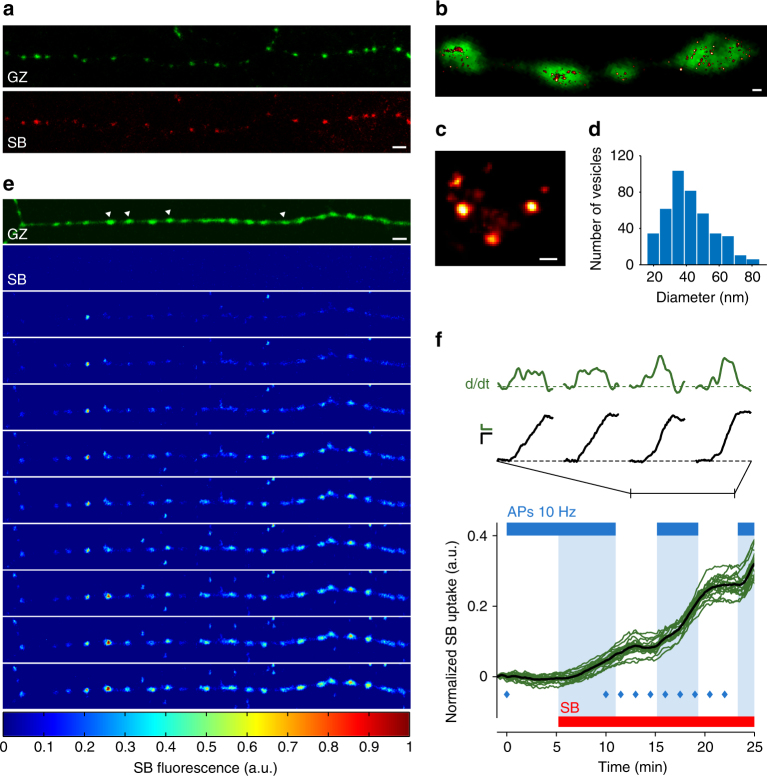
Synaptic expression of SynaptoZip and activity-dependent uptake of SB. **a** Presynaptic boutons expressing GZ (green) in acute hippocampal slices (Schaffer collateral, CA1). Slices were bathed with SB (red; SB 5 nM, 5 min, 24 °C, 30 mM isosmotic KCl). **b**–**d** Super-resolution microscopy of cultured CA3-CA1 hippocampal synapses. **b** Presynaptic boutons expressing GZ (green), with superimposed labeled synaptic vesicles, after spontaneous uptake of SB (hot red LUT; SB 5 nM; 60 min incubation, 37 °C). **c** Magnified view of synaptic vesicles loaded with SB. **d** Analysis of vesicles diameter following spontaneous uptake of SB (*N* = 428 SB-labeled vesicles from 24 synapses; 41.2 ± 16.2, mean ± SD). **e**, **f** Time-lapse imaging of SB uptake (SB 5 nM, 24 °C; color map, LUT at the bottom) in a group of boutons along a single axon (cultured hippocampal CA3–CA1 neurons), before and during trains of presynaptic action potentials (APs). In **f**, green lines are normalized SB uptake time series for *N* = 18 individual boutons (same experiment as in **e**; thick black line is the average SB uptake trace; blue diamonds correspond to time frames shown in **e**). The red bar refers to the presence of SB 5 nM in the bath, the blue bar indicates the stimulation epochs (trains of 10 AP at 10 Hz inter-leaved with 4 s pauses). Pale blue areas signal the occurrence of stimulation when SB is present. On top of graph, expanded traces to illustrate SB uptake (black lines; scale bar: 0.05 a.u. vs. 2 min) from 4 out of the 18 boutons (indicated by white arrowheads in **e**, GZ), and its rate (green lines, time derivative; scale bar: 0.05 a.u. h^−1^ vs. 2 min), during the second stimulation epoch. Notice that, despite the common trend, each synapse shows its own SB uptake dynamics. Scale bars: 4.11 µm (**a**, **e**), 400 nM (**b**), 40 nM (**c**)

The representative images of Fig. 2 illustrate that in both hippocampal cultures and acute slices, GZ expressing synapses are selectively labeled by bath exposure of SB. Indeed, as depicted in Fig. 2a (top), in acute hippocampal slices from animals transduced in the CA3 region, Schaffer collaterals labeling indicated a very precise colocalization between GZ-positive synapses and SB uptake sites. The VAMP-2-based design and the lumenal localization of the SB-binding site (Fig. 1a) suggest that the most likely mechanism of SB synaptic labeling relates to the exo-endocytosis of synaptic vesicles. In order to confirm this hypothesis, we used FM1–43, an established in vitro approach to study exo-endocytosis of synaptic vesicles^23^. Separately we evaluated SB localization inside synapses by super-resolution microscopy (ground-state depletion, GSD^24^). At FM1–43 preloaded GZ-expressing synaptic varicosities, evoked presynaptic activity was found to induce SB labeling and parallel unloading of FM1–43, thus indicating that both molecules use the exo-endocytic pathway (*N* = 6 experiments; an exemplar experiment is shown in Supplementary Fig. 4a–c). By super-resolution microscopy we found that the SB synaptic uptake resulted in the labeling of distinct vesicular structures (Fig. 2b–d), whose diameter distribution and average value (41.2 ± 16.2, mean ± SD; *N* = 428 vesicles from 24 synapses) were found to be fully consistent with those of synaptic vesicles^25^.

**Fig. 3:**
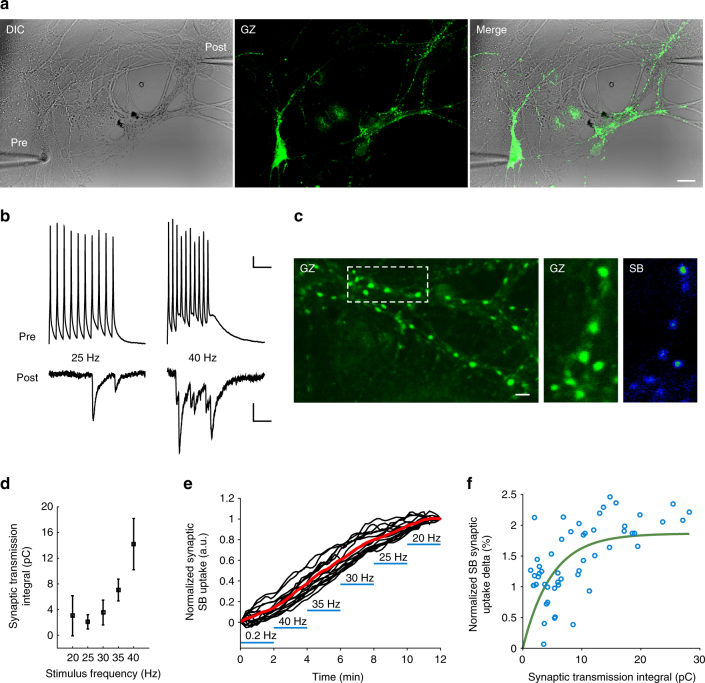
Synaptic transmission and SB uptake at hippocampal synapses. **a**–**f** Simultaneous electrophysiological and optical recording from a pair of synaptically connected hippocampal neurons (representative experiment from *N* = 5 pairs). **a** Images of the pre-synaptic and post-synaptic cells (left, DIC image; middle, in green GZ expression in the presynaptic cell; right, merge). **b** Evoked trains of action potentials in the presynaptic neuron (top traces) and the corresponding synaptic currents in the postsynaptic cell (lower traces) at two stimulation frequencies (25 and 40 Hz, 10 action potentials per train; trains applied every 5 s). **c** A subset of synapses, to illustrate reporter expression (GZ) and the effect of action potential trains on SB uptake (SB). **d** Analysis of the strength of synaptic transmission at different presynaptic stimulation frequencies (20–40 Hz trains as in **b**), indicating facilitation of EPSC amplitude. **e** Time-course of SB uptake (normalized for its final value) for *N* = 18 synapses belonging to the same axon at variable stimulation frequencies. **f** Percent increment of SB uptake for individual trains and synapses (synaptic charge and SB uptake were averaged over trains at the same stimulation frequency; same synapses shown in **e**). The green line is the fitting trace with a mono-exponential curve. Notice that the correlation approaches a ceiling for very high postsynaptic current values. Scale bars: 20 µm (**a**), 10 mV or 25 pA, 100 ms (**b**), 5 µm (**c**)

We then investigated the relation between the level of circuit activation and SB synaptic uptake. Synaptic uptake of SB was monitored at synapses belonging to individual axons of GZ expressing cultured hippocampal neurons, which were stimulated and recorded from in the whole-cell mode (WC; *N* = 11 independent experiments). As depicted in the representative experiment shown in Fig. 2e, f, the elicitation of action potentials (10 Hz trains, 10 APs each, 4 s inter-train intervals) induced clear synaptic uptake at GZ-positive boutons in the presence of extracellularly applied SB-Alexa647 (pale-blue shaded areas in Fig. 2f; SB 5 nM; 24 °C; *N* = 18 boutons; see also Supplementary Movie 2). Without stimulation, spontaneous synaptic uptake remained fairly low. This is clearly indicated by the drop in the uptake rate when the elicitation of action potentials was interrupted (white-shaded areas in the presence of SB in Fig. 2f). The activity-dependent nature of SB synaptic uptake was further confirmed in experiments where neurons were bathed in tetrodotoxin (TTX; 1 µM), which was found to strongly reduce SB uptake rate (data not shown). Importantly, application of SB to the extracellular medium or its AP-dependent uptake did not perturb action potential firing and the electrophysiological synaptic behavior. The latter conclusion arises from the observed stability of evoked synaptic currents (Supplementary Fig. 5a, b), as well as amplitude and frequency of miniature events (mini frequency and amplitude before and during SB, *p* = 0.31 and *p* = 0.12, respectively; TTX, 1 µM; Supplementary Fig. 5c, d). Since synapses integrate pre-synaptic and post-synaptic compartments to evaluate the relation between the strength of transmission and the amount of SB uptake, we conducted a series of dual electrophysiological recordings from synaptically connected pairs of neurons (*N* = 5 pair recordings; Fig. 3a). To modify the amount of transmitter release and therefore SB uptake, we varied the presynaptic AP stimulation frequency (0.2–40 Hz). As evident from the representative experiment shown in Fig. 3a–f, the increase in stimulation frequency induced a clear facilitation of excitatory postsynaptic currents (EPSCs) (Fig. 3b, d) and larger EPSCs were found to correlate with higher increments in synaptic SB uptake (Fig. 3e). This relationship diverges from a linear approximation just for very large synaptic currents, indicating a reduced sensitivity for strong release regimes. In summary, the vesicular localization of SB and the uptake dependence upon presynaptic activation strongly support the validity of SynaptoZip as a tool to measure the degree of synaptic transmission.

### SynaptoZip reports evoked activation of brain circuits

**Fig. 4:**
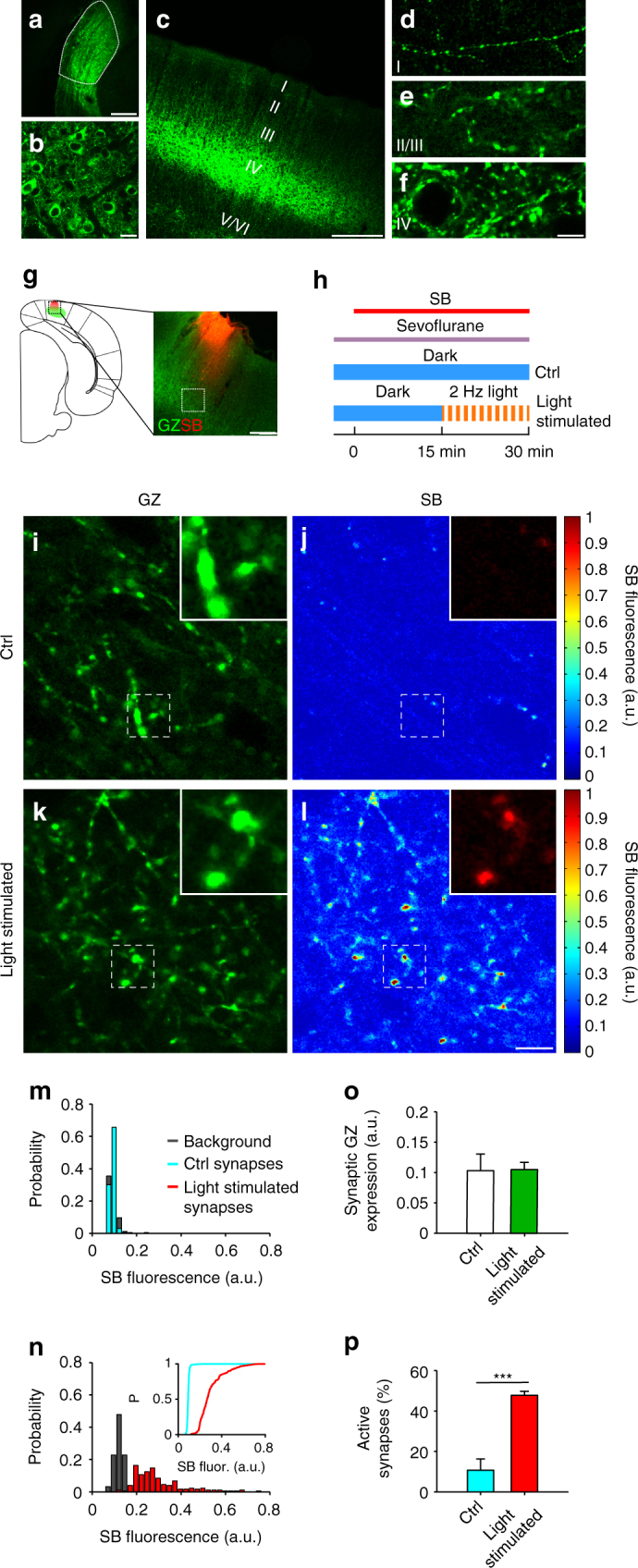
Reporting the activation of thalamo-cortical synapses in vivo. **a** Visual thalamus (dLGN, dashed area) transduced by GZ lentiviral vector. **b** magnified view of transduced dLGN. **c** GZ-expressing synapses in V1 cortex. **d**–**f** Magnified views of layers I, II–III, and IV showing GZ-expressing presynaptic boutons. **g** Illustration (left) and merged fluorescent images (right) of the V1 cortex with sites of GZ expression (green) and SB injection (red; dashed square indicates a typical field for SB uptake analysis; see also Supplementary Figs. 6, 8). **h** Time flowchart showing SB perfusion, sevoflurane anesthesia, and dark or dark/light exposure visual stimuli. **i**–**l** V1 layer IV from control animals kept in the dark (Ctrl; **i**, **j**) or exposed to light-pulses (light stimulated; **k**, **l**), showing GZ expression (**i**–**k**) and SB uptake (**j**–**l**). Insets are magnified areas from dashed boxes. Color bars on the right are fluorescence LUTs. **m**, **n** SB fluorescence histograms for unstimulated control (**m**; cyan bars; *N* = 233 synapses and *N* = 93 neighboring non-GZ^+^ areas, *p*_ctrl-background_ = 0.45, KS test) and light stimulated (**n**; red bars; *N* = 177 synapses and *N* = 92 neighboring non-GZ^+^ areas, *p*_light-background_ < 0.0001, KS test) GZ-expressing synapses, and for the corresponding background fluorescence (gray bars; same experiments as **i**–**l**). Inset **n**, cumulative distributions of synaptic SB uptake for data set in **m**, **n** (*p*_light-ctrl_ < 0.0001, KS test). **o**, **p** Population data from control and light-stimulated animals for synaptic GZ expression (**o**; *N* = 5, *p* = 0.93; two-samples permutation test) and activity (**p**; *N* = 5, *p* < 0.005; two-samples permutation test). Scale bars: 245 μm (**a**, **c**, **g**), 10 μm (**b**, **d**–**f**, **i**–**l**)

**Fig. 5:**
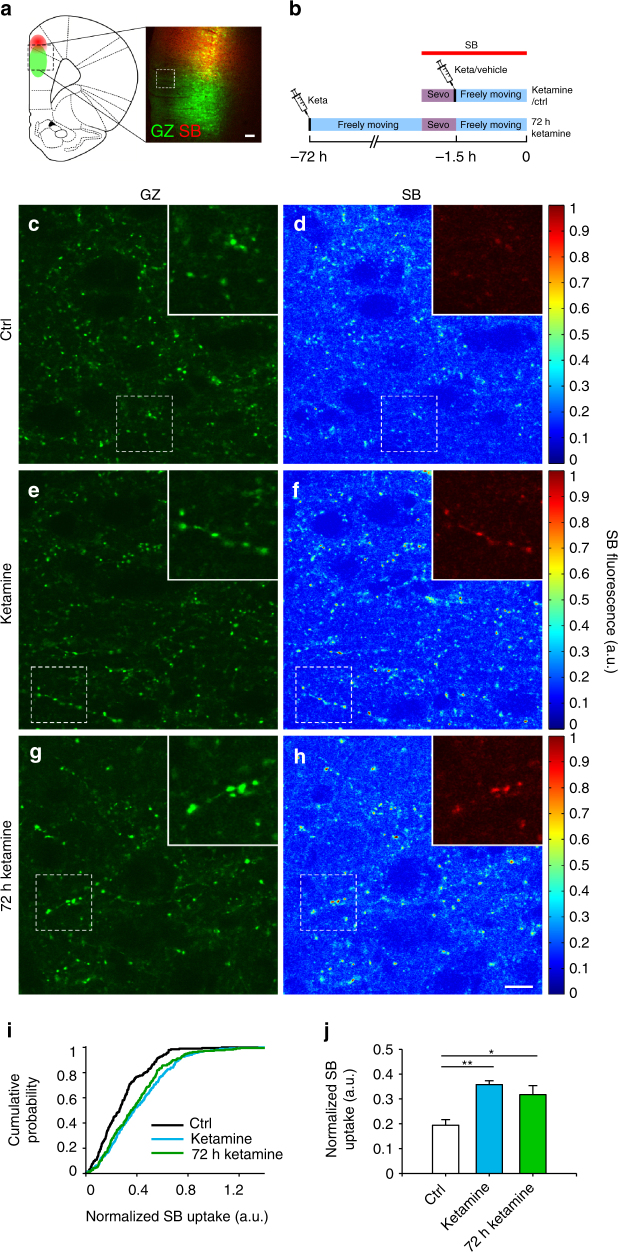
SynaptoZip reveals that ketamine induces a rapid and sustained increase in the activity at mPFC synapses. **a** Illustration (left) and merged fluorescent image (right) of the mPFC with the sites of GZ expression (green) and SB injection (red; dashed square indicates a typical field for SB uptake analysis; see also Supplementary Figs. 6, 8). **b** Time flowchart indicating SB delivery, ketamine treatments, and sevoflurane anesthesia (Sevo). **c**–**h** GZ expression (**c**, **e**, **g**) and SB uptake (**d**, **f**, **h**) in mPFC layer II–III from control (ctrl; **c**, **d**), ketamine (**e**, **f**), and 72 h ketamine (**g**, **h**) groups. Insets: magnified areas showing synapses. Fluorescence LUTs on the right. **i** Cumulative distributions of SB uptake from GZ-expressing synapses (*N*_ctrl_ = 216, *N*_ket_ = 275, *N*_72hket_ = 227; *p*_ctrl-ket_ < 0.0001, *p*_ctrl-72hket_ < 0.0001; *p*_ket-72hket_ = 0.35; KS test, B-H correction; same experiments as in **c**–**h**). **j** Population data for normalized SB synaptic uptake from control and ketamine treated groups (*N* = 6 rats for each condition; *p*_ctrl-ket_ < 0.01; *p*_ctrl-72hket_ < 0.05; *p*_ket-72hket_ = 0.32; two-samples permutation test, B-H correction; see also Supplementary Fig. 12). Scale bars: 100 μm (**a**), 10 μm (**c**–**h**)

The clear functional signal obtained from synapses in acute brain slices (Fig. 2a) highlights that SB diffuses inside the brain tissue, reaching the synaptic cleft. This prompted us to test this method in the living brain. SynaptoZip was expressed in rat V1 cortex and medial prefrontal cortex (mPFC) using viral vectors, followed by the local nano-rate delivery of SB through a glass micropipette (Supplementary Fig. 6a–d). Thanks to the long-term stability of the SB–GZ pair (Fig. 1f), the amount of SB uptake could be determined ex vivo, thus grasping signals even from boutons that could not be reached with the today available optical techniques. We found clear evidence for in vivo SB uptake at synapses (Figs. 4, 5). The efficacy of this approach likely relates to the small M.W. and therefore diffusibility of the SB peptide, since larger M.W. tracers (anti-Myc antibodies targeted to the C-terminal luminal epitope of SZ) did not work in similar conditions (Supplementary Fig. 7a–e). Great care was taken to improve the assay reproducibility, with a precise location and timing of SB application, and by selecting the most appropriate region for image acquisition based on SB tissue distribution profile (Supplementary Fig. 8a–c, e; see Methods).

We began by exploiting the rat primary visual cortex (V1), where circuit activity can be easily controlled by light (Supplementary Fig. 9a–e). With this aim in mind, the visual thalamus (LGN) was stereotaxically transduced in its dorsal portion (dLGN; see methods for details), resulting in clear GZ expression in thalamic neurons and ipsilateral V1 thalamo-cortical synapses (Fig. 4a–f). These eGFP fluorescing presynaptic varicosities were located in layer IV and to a smaller extent in deeper as well as in more superficial cortical layers of the V1 cortex (Fig. 4c–f). In these experiments, after GZ cortical expression, SB was delivered into the V1 superficial layers (I–II) (Fig. 4g, Supplementary Fig. 6a, b).

To test whether SB uptake could be induced by evoked activation of thalamo-cortical synapses, these animals were either exposed to visual stimulation with brief light pulses (light stimulated; pulse width 250 ms, intensity 340 µW/cm^2^, at 2 Hz for 15 min) or kept in the dark for the same length of time (ctrl; Fig. 4h). The light-stimulation protocol was devised in a series of preliminary experiments aimed at eliciting reproducible responses with a good balance between ON and OFF phasic responses, to maximize synaptic cortical activation under sevoflurane anesthesia (Supplementary Fig. 9). In unstimulated animals, the SB functional signal obtained from GZ-positive boutons located in layer IV was weak, with few clearly SB fluorescing synapses, evidently reflecting a small degree of spontaneous tonic OFF activity (a representative experiment is shown in Fig. 4i, j). This is consistent with the strong inhibition of spontaneous cortical activity by sevoflurane in V1 cortex^26^. Following repeated light ON-OFF stimulations, the synaptic SB uptake was augmented, with a clear increase in the number of SB fluorescing thalamo-cortical layer IV GZ-positive boutons (a representative experiment is shown in Fig. 4k, l; see also Supplementary Movie 3). Our experimental protocol and analysis approach allowed selecting FOVs for synaptic uptake analysis located on the flat portion of SB spatial concentration profile (600–800 µm from microperfusion sites) resulting in low extracellular SB concentration variability and comparable levels in non-synaptic areas among the different experimental groups (Supplementary Fig. 8d; see Methods). Fluorescence histograms obtained from the above experiments clearly indicate that in the absence of stimulation the synaptic SB uptake is not distinguishable from tissue background (Fig. 4m; *N* = 233 synapses and *N* = 93 neighboring non-GZ^+^ areas, *p*_ctrl-background_ = 0.45, KS test). On the contrary, ON-OFF light stimulation markedly displaced the uptake distribution from background (Fig. 4n; *N* = 177 synapses and *N* = 92 neighboring non-GZ^+^ areas, *p*_light-background_ < 0.0001, KS test), as well as from the unstimulated condition (Fig. 4n inset; *p*_light-ctrl_ < 0.0001, KS test). This indicates a sharp increase in the number of active sites by ON-OFF light stimulation. Across different experiments, while the synaptic expression of the reporter GZ was found to be comparable (Fig. 4o; GZ expression: control, *N* = 5 rats, 0.1 ± 0.03; light stimulated, *N* = 5 rats, 0.11 ± 0.01; mean ± s.e.m.; *p* = 0.93; two-samples permutation test), the average number of active thalamo-cortical synapses was significantly increased by the ON-OFF light stimulation (Fig. 4p; % synapses with SB uptake above background: control, 10.7 ± 5.5%, *N*_control_ = 5 rats; light stimulated, 47.7 ± 1.9%, *N*_light-stimulated_ = 5 rats; mean ± s.e.m.; *p* < 0.005, two-samples permutation test; threshold for synapse selection 2 SD of background noise). Overall, this suggests that GZ can be effectively used to report stimulus evoked activation of brain circuits occurring in vivo.

### SynaptoZip detects enduring activity changes by ketamine

To further test the applicability of this approach in freely moving animals, we examined the effect of ketamine on the mPFC. Previous micro-dialysis experiments have provided evidence that a single sub-anesthetic dose of ketamine induces neurotransmitter accumulation in the extracellular space^27,28^, although the involvement of synaptic exocytosis and the contribution of the intrinsic mPFC circuitry are not established. Prelimbic mPFC of adult rats was stereotaxically transduced with GZ in layer V (Fig. 5a), resulting in the appearance of eGFP fluorescing neurons and axonal synaptic boutons belonging to the local mPFC circuitry (layers I–VIa; Supplementary Fig. 10). After 2–3 weeks from transduction, a bolus of SB was micro-perfused at low rate, ∼700 µm above the GZ–LV injection site (Fig. 5a, Supplementary Fig. 6c). During this SB perfusion epoch (30 min), run under sevoflurane anesthesia, SB efficiently spread and accumulated in the mPFC (Supplementary Fig. 6d), with negligible synaptic uptake (Supplementary Fig. 11a, b). At the end of SB delivery epoch, animals were woken up from anesthesia and left freely moving for 90 min, set as the observation window for synaptic activity. To test for the effect of ketamine, we evaluated synaptic uptake at early and late (72 h) time points after the systemic injection of a single dose of ketamine (15 mg/kg; i.p. injection). In all experimental groups, including controls (vehicle injected), the 90 min freely moving epoch resulted in a clear SB uptake in a large fraction of GZ-expressing mPFC synapses (*N* = 6 rats for each condition; ctrl, 84%; ketamine, 92.4%; 72 h after ketamine, 91.5%; Fig. 5c–h), evidently a reflection of the strong spontaneous activity of local prefrontal circuits in the awake state^29^. Interestingly, this synaptic labeling was still detectable 7 days after SB uptake (Supplementary Fig. 11c, d), suggesting that, in this time frame, the previously generated synaptic GZ–SB complex remains stable. Compared to controls, both ketamine-treated groups showed a clear augmentation of synaptic SB uptake. At early and late time points from ketamine treatment, changes in synaptic uptake affected the entire range of activity levels, with an homogeneous shift of fluorescence cumulative distributions (Fig. 5i; *N*_ctrl_ = 216, *N*_ket_ = 275, *N*_72hket_ = 227; *p*_ctrl-ket_ < 0.0001, *p*_ctrl-72hket_ < 0.0001; *p*_ket-72hket_ = 0.35; KS test, Bonferroni–Holm (B-H) correction; data from same experiments presented in Fig. 5c–h). As for V1 cortex, in the mPFC areas where synapses were analyzed (600–800 µm from the SB injection site) the estimates of SB concentration in non-synaptic areas indicated that synapses were exposed to similar SB concentrations (Supplementary Fig. 8f). Population data from these experiments showed that at early times, compared to controls, ketamine almost doubled synaptic SB uptake, an activity change that is still observable 72 h after (Fig. 5j; *N* = 6 rats for each condition; ctrl, 0.194 ± 0.022; ketamine, 0.358 ± 0.015; 72 h after ketamine, 0.317 ± 0.037; mean ± s.e.m.; *p*_ctrl-ket_ < 0.01; *p*_ctrl-72hket_ < 0.05; *p*_ket-72hket_ = 0.32; two-samples permutation test, B-H correction; values corrected for variation in synaptic GZ expression, see Supplementary Fig. 12a–i and its legend for details about the selection of the uptake index used in mPFC experiments).

Ketamine is known to increase animal motility^30^, an effect that could indirectly drive mPFC activity by enhanced input from several sensory modalities. At early time points, ketamine was found to induce a clear increase in locomotion (Supplementary Fig. 13a–d, g, j, k), characterized by ataxic movements (Supplementary Fig. 13h, i). However, this behavior was apparently lost 72 h after ketamine injection (Supplementary Fig. 13e–k), while the potentiation of synaptic SB uptake persisted (Fig. 5i, j). Thus, the long-lasting change in mPFC circuit activity induced by ketamine is not explained by increased animal motility.

## Discussion

We have developed a novel synaptic activity reporter, which operates both in vitro and in vivo, whose integrated signal is durable and can be acquired not only online but most importantly by retrospective analysis. Signal integration arises from specific binding and trapping into the synaptic vesicle of the SynaptoZip partner SB, whose synaptic enrichment reflects the number of synaptic exo-endocytic events occurring during an experimental epoch. This method, which generates reliable activity estimates, fulfills the demand for a thorough post hoc analysis of brain synapses. Its application is cost-effective, and can reach synapses that are outside the grasp of standard optical imaging methodologies^19^, hence potentially usable to map the activity of the whole brain. Our approach is compatible with immunolabeling and possibly clearing methods (see for review^31^). Importantly, SynaptoZip could be combined with neuronal activity integrators^32–34^, to solve the functional contribution of upstream and downstream circuital elements.

Here, we have applied SynaptoZip to the V1 cortex, obtaining reliable estimates of light-evoked synaptic activity in the anaesthetized rat (Fig. 4). Indeed, like other exo-endocytosis indicators^10–17^, SynaptoZip generates a synaptic signal that depends on presynaptic action potential firing (Fig. 2), whose frequency is strongly modulated at thalamo-cortical synapses by phasic ON-OFF light stimulations (Supplementary Fig. 9). In the mPFC this approach was applied to reveal drug-induced changes in spontaneous synaptic activity in freely moving animals (Fig. 5). In these experiments we used ketamine, a drug that at sub-anesthetic doses exerts a rapid antidepressant effect, whose mechanism and site of action are still highly debated^28,35^. We found that the activity of the intrinsic mPFC synaptic network is rapidly enhanced by a single ketamine administration, a change that persisted for at least 72 h. This finding agrees with previous micro-dialysis experiments, which provided evidence for ketamine-induced glutamate accumulation in the extracellular space^27,28^. By use of SynaptoZip, we show that an enhanced vesicular release of glutamate at the level of layer V to layers II–III mPFC synapses is the most likely mechanism behind glutamate accumulation, which could induce and/or maintain subsequent plastic changes^36^ in the mPFC circuitry.

Regarding the design of the SynaptoZip–SB pair, we used the Velcro coiled-coil heterodimer^20^ for its very high-binding specificity and pair stability^20,21^, but also because the soluble peptide partner (SB) is small (3.4 KDa), thus favoring an efficient diffusion in brain extracellular spaces and into the synaptic cleft (Supplementary Fig. 7). In the future it might be interesting to evaluate alternative peptide pairs with different properties and binding affinities^37^. VAMP-2, SynaptoZip vesicular scaffold, has been previously used in other synaptic activity sensors^11,12,14^. VAMP-2 is the most abundant vesicular protein^38,39^, with a single transmembrane domain and a very short, presumably inert, intraluminal segment^40^. Conceivably, both factors have guaranteed the high level of vesicular expression found here, which is important to avoid a fast saturation of the integrated SB signal. In addition, because VAMP-2 is a ubiquitous synaptic vesicle protein, it is present at all synapses independently of their neurochemical phenotype, and SynaptoZip would be well integrated with the native fusion machinery.

As demonstrated here, our approach works well in vivo, although some methodological aspects would benefit from ad hoc future developments. At present, the major technical difficulty relates to the SB delivery phase into the brain tissue, a procedure that involves extreme accuracy during the experimental and subsequent analysis phase. Clearly, at a given stimulation rate, the cumulative index of synaptic activity at each bouton would depend upon the number of exo-endocytotic events along the experimental epoch, the experimental parameter that is important to extract, but also upon the average number of SB molecules successfully bound at each fusion event. The latter would be influenced by SB concentration in the extracellular space (Supplementary Fig. 12d–i), by fusion kinetics^16,18^ and its modality, but also by the average number of available binding sites at each individual vesicle that can be extracted by the expression signal. In our experimental conditions, the extracellular tracer concentration at regions selected for analysis was found to be fairly constant and comparable among the different groups (Supplementary Fig. 8d, f). Regarding the dynamics of fusion and retrieval of synaptic vesicles, known to display complex kinetics, a dissimilar coverage of the different modalities^41–44^ might occur. Such limitation might contribute to the shape of the relationship between SB uptake and synaptic transmission, with some hints of saturation at very high stimulation frequencies (Fig. 3f). In the future, it would be important to find alternative ways to deliver SB locally at synapses, but also to test smaller versions of these tracers. Despite these caveats, since the integrated output of SynaptoZip clearly correlates with the degree of synaptic activation (Figs. 2–4; Supplementary Fig. 4 and Supplementary Movie 2), this ensures that even at this stage, a reliable mapping of brain synaptic activity can be achieved, a result that cannot be obtained with any other presently available technique.

Concerning behavioral studies, if more effective ways for the chronic and timed-controlled delivery of SB to brain synapses were to be found, the analysis of time-dependent changes in synaptic activity in freely behaving animals would become feasible. Interestingly, the long-term stability of the vesicular SynaptoZip–SB complex for at least 1 week (Supplementary Fig. 11c, d) suggests that multiple chasing with different SB fluorescent versions could track the fate of synapses and synaptic vesicles, thus helping to address important queries in behavioral neuroscience but also in neurobiology^45^, and in the field of circuit plasticity^36^. This technology could also be used for the selective delivery of molecules to active terminals, to tag or remove circuits involved in specific behaviors. In the future, following ad hoc labeling of SB with radioactive isotopes or paramagnetic molecules, our approach could be adapted for in vivo imaging by animal PET and fMRI. Since antibodies targeting natural intraluminal vesicular epitopes^46,47^ cannot be applied as in vivo functional probes (Supplementary Fig. 7), we envisage that the search for small ligands for native synaptic vesicle intraluminal epitopes may be an important and rewarding future avenue, capable of extending our approach to naïve animals.

## Methods

### Research and animal procedures

Research and animal care procedures were approved by our Institutional Animal Care and Use Committee for Good Animal Experimentation in accordance with Italian MIUR code of practice for the care and use of animals for scientific purposes (IACUC numbers: 576, 541, and 543). Experiments were performed on Sprague Dawley male rats (150–350 g). Animal group allocation was known by the investigators. All efforts were devoted to minimize animal’s distress, pain, and suffering during the entire course of the experimentation. All animals were caged with free access to food and water ad libitum and were exposed to 12 h light/dark cycles at 23 °C constant room temperature.

### Constructs and expression vectors

SynaptoZip (SZ) was made starting from a cDNA clone in pBluescript KS provided by Elferink and colleagues^48^ carrying the *Rattus norvegicus* Synaptobrevin-2/VAMP-2 CDS and 3′UTR. A Bsu36I fragment containing 231 bp downstream the natural stop codon (TAA, nucleotides 431–433 in Ref. Seq. NM_012663.2) was excised leaving a unique Bsu36I site that abuts the stop codon. The latter was mutated to a Serine codon (TCT) and a synthetic Bsu36I fragment (174 bp) was inserted in frame that codes, in the order, SVPEG (an adapter) and KGVEPKTYCYYSS (a spacer^49^), AQLEKELQALEKENAQLEWELQALEKELAQ (Acid-p1^20^), EQKLISEEDI (c-Myc tag), ending with an ad hoc stop codon (TGA). The remaining 3′UTR (1300 bp) was left intact and in place. For the expression vector of the non-fluorescent variant SynaptoZip (SZ) a HindIII-SacI fragment was transferred into pBeta-Actin, kindly provided by Andrew Matus. The fluorescent variant eGFP-SynaptoZip (GZ) was obtained by in-frame fusion to eGFP at the EcoR1 site of pEGFP-C2 (Clontech); it comprises 13 foreign codons from the vector MCS and lacks the first VAMP-2 codon. For insertion into the lentiviral (LV) transfer vector, kindly provided by L. Naldini^50^, an Age1-Sal1 fragment containing GZ was used to replace the pre-existing eGFP, resulting in GZ-LV transfer vector. LV particles were produced as previously described^50^ and stock titers (~10^9^ TU ml^−1^) determined. All constructs were confirmed by DNA sequencing (Eurofins Genomics, Italy). SB peptide was produced by synthesis (CGGAQLKKKLQALKKKNAQLKWKLQALKKKLAQ; JPT Peptide Technologies GmbH, Berlin, Germany) and fluorescent dyes were conjugated to the terminal Cys (Alexa Fluor Dyes®: 488, 568, 647; Thermo Fisher Scientific).

### Experiments with Hela cells

Hela cells (ATCC^®^ CCL-2™; LGC Standards S.r.l., Italy; STR authenticated), regularly tested to be mycoplasma free, were grown on glass coverslips (25 mm diameter) or plastic petri dishes (10 cm diameter) at 37 °C, in 5% CO_2_ humidified incubator in DMEM supplemented with 10% (v/v) FCS, GlutaMax, and antibiotics. For SZ expression, cultures were transfected with vector DNA using Lipofectamine 2000 (Invitrogen) or transduced with GZ-LV vector pre-diluted in culture medium (final titer for transduction in vitro: ~10^6^ TU ml^−1^). Incubation with SB (0.1–100 nM) was run either in DMEM at 37 °C 5% CO_2_ or in oxygenated Tyrode solution at 23 °C. For long-term experiments, DMEM was supplemented with 1% FCS (v/v). For fixed samples, at the end of incubation, cells were extensively washed first with cold Tyrode solution containing 1% BSA, followed by PBS and PFA 4% (30 min, 4 °C).

### Western blot analysis

Lysates from Hela cells (50 µg) and neurons (20 µg) were subjected to SDS-PAGE (14%) then transferred onto nitrocellulose filters (0.22 µm). Protein bands were detected by fluorescent SB, Mouse anti-c-Myc monoclonal 9E10 (Abcam), Mouse anti-VAMP2 monoclonal (Synaptic System) or Rabbit anti-GFP (Merck Millipore) primary antibody, the latter three followed by the respective peroxidase-conjugated secondary antibody (Biorad). Chemiluminescence was with Hyperfilm ECL (GE-Healthcare Bio-Sciences), signal acquired by Personal Densitometer (GE-Healthcare Bio-Sciences). For detection with SB, filters were incubated (5 nM SB-Alexa647 in blot solution, for 1 h at 24 °C) then washed (blot solution, five washes, 10 min each), and fluorescence detected by Typhoon800 (GE-Healthcare Bio-Sciences).

### Neuronal cultures

Primary postnatal neuronal cultures were prepared from Sprague Dawley rats (2–5 days old) as previously described^46,51^. In brief, P2–P5 rats were rapidly decapitated and hippocampi dissected out. Dissociated neurons were grown on poly-L-ornithine (10 µg ml^−1^) and Matrigel (1:50 dilution; Becton, Dickinson and Company, NJ) coated 35 mm petri dishes or glass coverslips (25 mm). Neurons were maintained in a CO_2_ incubator (5% CO_2_, 37 °C; Heraeus Instruments GmbH, Hanau, Germany) using a modified minimum essential medium with Earle’s salts (Gibco®, Life Technologies) in 5% dialyzed FCS (Gibco®, Life Technologies). Every 3 days one-third of the culture medium was replaced with fresh medium supplemented with cytosine β-D-arabinofuranoside (Ara-C; 2.5–5 µM) to prevent excessive glial cells proliferation. For GZ expression, neurons were transduced with GZ-LV vector pre-diluted in culture medium 1 h after plating.

### Acute hippocampal slices

Acute hippocampal slices were obtained from Sprague Dawley rats (150–200 g) previously transduced (2 weeks before) with GZ-LV in the right hippocampus CA3 (stereotaxic coordinates: 3.8 mm mediolateral, ML, from the sagittal axis; −3.55 mm rostrocaudal, RC, from bregma; 3.8 mm dorsoventral, DV). For brain acute slice preparation, after a lethal injection of thiopental (50 mg i.p.; RotexMedica, GMBH, Germany), rats were intracardially perfused with ice-cold, modified Ringer solution adapted for cutting (119 mM NaCl, 2.5 mM KCl, 1 mM CaCl_2_, 3 mM MgSO_4_, 26.2 mM NaHCO_3_, 1 mM NaH_2_PO_4_, 11 mM D-glucose) bubbled with 95% O_2_ and 5% CO_2_, and containing 5000 IU l^−1^ heparin (Pharmatex, Milano, Italy). After decapitation, brains were quickly removed and transferred to ice-cold modified Ringer. After dissection of the right hippocampus, transverse slices (450 μm thick) were cut using a tissue chopper (Stoelting, Wood Dale, IL), submerged in the above solution adapted for recording (2 mM CaCl_2_ and 1.3 mM MgSO_4_), warmed at 33.6 °C for 1 h and then maintained at room temperature up to 6 h. For SB uptake experiments, acute slices were placed on the slice recording chamber positioned under the microscope and extracellularly perfused with the above recording solution (24 °C, bubbled with 95% O_2_ and 5% CO_2_). SB (5 nM) was directly added to the extracellular solution, modified by the addition of 30 mM KCL (isosmotic).

### Electrophysiological recordings and FM1–43 experiments

Cultured neurons were used for electrophysiological experiments 10–21 days after plating. All electrophysiological recordings were obtained using WC patch clamp (current-clamp or voltage-clamp configuration) and neurons were continuously superfused (1–2 ml min^−1^) at room temperature (24 °C) with a Tyrode solution containing (in mM): 119 NaCl, 5 KCl, 2 CaCl_2_, 2 MgCl_2_, 25 HEPES, and 30 D-glucose (osmolarity 305 mOsm, pH 7.4). For miniature currents recordings, this solution was supplemented with the voltage-gated sodium channel blocker TTX (1 µM; Latoxan, Valence, France). For SB uptake experiments in current-clamp, bath was supplied with the NMDA receptor blocker D-2-amino-phosphonovalerate (APV; 25 µM; Tocris Cookson, Bristol, UK) and the AMPA receptor antagonist 6-cyano-7-nitroquinoxaline-2,3-dione (CNQX; 10 µM; Tocris Cookson, Bristol, UK) to inhibit network activity. Patch pipette electrodes (resistance 5–10 MΩ) were filled with an intracellular solution containing (in mM): 110 D-gluconic acid, 5 MgCl_2_, 10 NaCl, 0.6 Ethylene glycol-bis(2-aminoethylether)-N,N,N,N-tetraacetic acid (EGTA), 2 ATP, 0.2 GTP, and 49 HEPES (pH adjusted to 7.2 with CsOH; osmolarity 290 mOsm). When testing evoked transmission between neuron pairs CNQX was not applied to the bath and the pipette patching the presynaptic neuron was filled with a K-gluconate solution containing (in mM): 120 K-D-Gluconate, 20 KCl, 2 MgCl_2_, 0.2 EGTA, 10 HEPES, 0.2 GTP-Na_2_, 2 ATP-Na_2_ (pH adjusted to 7.2 with KOH; osmolarity 290 mOsm), while QX-314 was added to the pipette patching the postsynaptic neuron (1.5 mg ml^−1^, Tocris Cookson, Bristol, UK). Current-clamp recordings were obtained with an EPC7 amplifier (HEKA Elektronik, Lambrecht/Pfalz, Germany; membrane potential maintained at ~ −75 mV to reduce spontaneous action potential firing). Evoked and miniature currents were acquired in voltage-clamp (holding potential −70 mV; Axopatch 200B or 200A amplifier; Axon Instruments, Foster City, CA). Membrane and series resistances were constantly monitored by applying 2–5 mV depolarizing pulses (recordings with series resistance higher than 20 MΩ were discarded). All traces were filtered at 2–5 kHz and digitally acquired at 20 kHz using a 16-bit analog-to-digital interface (ITC-18; HEKA Elektronik, Lambrecht/Pfalz, Germany) controlled by custom acquisition software (Labview and C/C++). Analysis of recordings was performed with custom code developed in Matlab (Mathworks; miniature detection algorithm as previously described^51^). In vivo recordings of visual evoked potentials (PEVs) were performed on Sprague Dawley rats (300–350 g) under general anesthesia (sevoflurane 3.5%) by measuring electro-cortical activity through screws implanted in the skull^26^. For FM1–43 experiments, synapses were labeled for 1–2 min with 10 μM FM1–43 (Molecular Probes—Thermo Fisher) dissolved in modified Tyrode solution (isotonic 90 mM KCl; 10 μM CNQX; APV 25 μM). After 15 min of perfusion with a washing Tyrode solution (10 μM CNQX; APV 25 μM; TTX 1 μM), unloading was obtained in a standard Tyrode solution (10 μM CNQX; APV 25 μM) by field stimulation using two parallel Platinum electrodes (constant current stimulation, 40 mA/cm; 10 APs trains at 10 Hz, 4 s inter-train interval).

### Anesthesia and stereotaxic surgery

Anesthesia was induced by inhalation of 5% sevoflurane in an induction chamber (Abbvie, North Chicago, IL, USA) until the animal was unconscious, based on loss of the righting reflex. Anesthesia was maintained by injection of ketamine/xylazine (50 mg/kg; Ketavet 100® Veterifarm; xylazine 5 mg/kg, Rompun®, Bayer) or, alternatively, by 3.5% sevoflurane insufflation into the intubated, mechanically ventilated animal^26^ (tidal volume 6 ml; respiratory rate 70–80 breaths min^−1^). Anesthetized animals were then injected with dexamethasone (0.2 mg/kg s.c.; Dexamethasone Phosphate, Hospira Srl) and gentamycin (1.5 mg/kg i.p.; Gentamycin Sulphate, Italfarmaco), and an eye ointment (Xantergel®, SIFI S.p.a) was applied bilaterally to prevent drying. For stereotaxis the skull was disinfected with 2% Chlorhexidine (CITROclorex 2%®, Esoform) and Betadine (Eso jod 10%®, Esoform). A custom-made stereotactic apparatus^52^ was used for LV or SB delivery into brain. For injection, glass micropipettes (60 μm tip diameter; borosilicate, VWR International) were front-filled and connected with a P10 Teflon tube to a 50 µL Hamilton micro-syringe pre-filled with mineral oil, operated by a motor-driven micro-perfusion system (100 nl min^−1^; Harvard Apparatus Pump 11 Elite). At the end of stereotaxic delivery, sutures were placed under anesthesia, the animal returned to cage and the recovery and health state monitored. For viral transduction, LV particles in PBS (~10^9^ TU ml^−1^ LV; 6 µl of PBS solution supplemented with 0.3 M mannitol^53^) were injected. Before pipette withdrawal, the micro-pipette was left in place for 10 min to allow for diffusion of LV particles. For visual experiments, LV particles were injected into the right dLGN (3.65 mm ML, −4.35 mm RC from bregma, 5.20 mm DV) under ketamine/xylazine anesthesia. For mPFC experiments, LV particles were delivered into the mPFC (0.65 mm ML, +3.5 mm RC, 3.5 mm DV coordinates) under sevoflurane anesthesia.

### In vivo SB uptake experiments

A balance between subjects design without randomization was adopted for all in vivo SB uptake experiments. In most experiments, two animals were tested a day and their temporal order was reversed the next session (for example, control animal before/after light stimulated animal). For experiments on the visual system, all phases were run with animals fully anaesthetized as described above. 2–3 weeks from viral transduction, in the preparatory phase, the animal was blindfolded bilaterally in order to prevent exposure to ambient light during surgery and micro-pipette placement. Stereotaxic coordinates for SB delivery were: V1, 2.85 mm ML; −7.35 mm RC; 0.8 mm DV. For these experiments we used SB-Alexa647, 13.3 μM dissolved in standard Tyrode solution (30 min; 100 nl min^−1^). During SB perfusion control animals were maintained in complete darkness. Light-stimulated animals were initially kept in complete darkness (15 min), then their left eye was unblinded and light pulses were administered for the subsequent 15 min. Light stimulation was delivered using a white LED matrix placed at a distance of 1.2 cm from the eye (250 ms pulse duration; 340 μW irradiance; 2 Hz stimulation rate). For mPFC experiments, animals fully anaesthetized as described above were locally perfused with a bolus of SB (SB-Alexa647, 1.9 μM dissolved in standard Tyrode solution; 30 min; 100 nl min^−1^) at coordinates 0.65 mm ML, +3.5 mm RC, 2.8 mm DV; after micro-pipette removal and re-suturing (see above). Animals were waken up and left freely moving in cages for 1.5 h. For drug treatments, ketamine (15 mg/kg) or vehicle were i.p. administered either immediately after the end of micro-perfusion of SB-Alexa647 (animal was still under anesthesia), or 72 h before micro-perfusion of SB-Alexa647. At the end of perfusion, animals still under general anesthesia were sacrificed with a lethal dose of Thiopental (Thiopental, Vuab-Pharma) and then intracardially perfused with cold saline supplemented with heparin (5000 IU l^−1^), followed by PFA 4% in 120 mM phosphate buffer (pH 7.4 at 4 °C). The brain was removed, submerged in fixative O/N (4 °C), embedded in 4% agar. 35–40 µm thick slices were cut with a vibratome (VT1000S, Leica, Germany), when required processed for immunofluorescence, then mounted onto glass slides (Fluorsave, EMD Millipore). For detection of PSD95A, we used the method by Schneider et al.^54^ on unfixed 15 µm thick slices (Cryostat sectioning; CM 1850, Leica, Germany).

### Behavioral analysis

For behavioral experiments a balanced within subjects design without randomization was adopted. Rats were placed in a behavioral cage and their motility monitored after their awaking from sevoflurane anesthesia, in conditions fully matching in vivo SB experiments. The behavioral cage was composed of the rat’s home cage bottom (38 × 22 cm^2^) and a custom-built roof hosting a digital camera for video recording (1280 × 720 pixel^2^ resolution, 25 fps, NoIR v2 camera controlled by Raspberry Pi 3, Raspberry Pi Foundation). Food and water were always provided during sessions (water dispenser in the top-right corner; room temperature 23 °C, low light illumination). Rats were subjected to four video recording sessions each lasting 90 min (day 0: preconditioning to behavioral cage; day 1: control recording; day 2: recording after ketamine administration; day 5: recording 72 h after ketamine administration). Ketamine was administered during anesthesia (i.p., 15 mg/kg). Tracking and analysis of rat movement were performed using custom software (Python and Matlab). A pause was considered as a period of time where rat barycenter remained confined for more than 10 s in a circular area (2 cm radius). The central area crossing index used an area at the center of the cage with dimensions 19 cm × 11 cm.

### Immunofluorescence

Cells and tissue samples were fixed (4% PFA), quenched (0.1 M glycine, 120 mM phosphate buffer, pH 7.4, 30 min, 4 °C), and permeabilized in blocking buffer (0.4% Saponin for cell cultures, 3% Triton X-100 for brain slices; 120 mM phosphate buffer, 1% BSA, pH 7.4, 1 h, 4 °C). Samples were then incubated with primary antibody (1:100–1:300 v/v in blocking buffer; 1–2 h at 23 °C or O/N at 4 °C) followed by secondary antibody (1:200 v/v in blocking buffer, 1–2 h at 23 °C). Samples were washed with blocking buffer (4 °C), rinsed in PBS, and mounted (Fluorsave, EMD Millipore). Primary antibodies used: anti-PSD95 (mouse IgG2a, monoclonal; clone 7E3-1B8, Merck Millipore), anti-c-Myc (mouse monoclonal clone 9E10; Abcam, Cambridge, UK), anti-GAD 65 (mouse IgG2a, monoclonal; clone GAD-6; Sigma), anti-p38 synaptophysin^48^ (mouse IgG1, monoclonal; clone 7.2; Synaptic Systems, Goettingen, Germany), anti-GluR-1 (rabbit polyclonal; generated in house), anti-SNAP25 (rabbit polyclonal; cat. 111002, Synaptic Systems), anti-p65 synaptotagmin (rabbit and goat polyclonal^46^), anti-GFP (rabbit polyclonal; cat. A6455, Thermo Fisher Scientific). Secondary antibodies used: Donkey anti-Mouse, anti-Rabbit, and anti-Goat AlexaFluor488/568/647 (Jackson ImmunoResearch).

### Image acquisition and data analysis

All fluorescence images (excluding super-resolution) were obtained using a confocal microscope (LSM510, Zeiss) equipped with 488, 543, and 633 nM laser lines and standard filter sets for FITC, TRITC, and CY5 excitation/emission spectra. In preliminary experiments we evaluated the range of acquisition parameters (pinhole size, laser power, and detector gain) best suited to each experimental condition, in order to favor signal to noise ratio while preserving linearity and avoid signal saturation. Typhoon800 data acquisition was performed on all brain sections (Supplementary Fig. 6) to identify the SB injection site. At the beginning of confocal imaging acquisition, low-magnification scanning (10× objective) was carried out on slices to evaluate GZ expression and locate the highest SB intensity spot and select the appropriate areas for the high-magnification acquisition step (63× objective; Supplementary Fig. 8). No relevant information about SB uptake by synapses could be discerned at low magnification. The high-magnification step to acquire images from synaptic areas (as well as later selection of mPFC axons) was performed using the GZ signal as reference, so that no information about the functional signal (SB uptake) was known by experimenter. Image analysis was performed using either ImageJ (NIH) or custom code developed in Matlab (Mathworks). All numerical analyses were then performed in Matlab (Mathworks) or Origin (OriginLab).

For experiments with Hela cells, cell-contours were segmented based on GZ fluorescence (or on SB-Alexa647 fluorescence when expressing SZ lacking eGFP). Hela vesicles were identified by the Particle Analyzer plugin of ImageJ using thresholding parameters based on the average and SD of vesicle size, roundness, and fluorescence. For Hela vesicles fluorescence thresholding, background noise was measured in vesicle-free regions near the plasma membrane using a circular ROI with similar radius and fluorescence thresholds were then set to mean background fluorescence plus 2 SD. For cultured neurons experiments, confocal time-lapse recordings (interval 10 s) were filtered by median and Kalman filters (equalized for producing color maps). Synapses belonging to the same axon were manually selected on the GZ channel and automatically segmented in each frame. SB and GZ fluorescence were averaged at each synapse and their ratio taken as normalized SB uptake. The time series produced was filtered (moving average on four samples) after removing the initial baseline (no SB in the bath). For super-resolution localization of SB uptake in synaptic vesicles, we used a GSD microscope (SR GSD 3D, Leica Microsystems, Buffalo Grove, IL), the PALMsiever Matlab toolbox was then used to generate images from localization lists (kernel density estimation, pixel size set to 4.12 nm). Standard Matlab functions were utilized for circle segmentation and measurements of vesicle diameters (synapse contours were manually traced using the eGFP low-resolution image).

For in vivo experiments, synapses were automatically identified and segmented based on GZ fluorescence. A few animals were not included in the analysis when GZ viral transduction was below detection (lack of a significant eGFP signal in neuronal somata at the site of transduction; *N*_excluded_/*N*_tot_: V1, 5/15; mPFC, 6/24). Due to discrepancies in tissue morphology, different approaches were used for V1 and mPFC experiments. In the former case, synapses were segmented using a custom algorithm based on image thresholding, Voronoid tessellation and then selected based on shape and fluorescence intensity (threshold: 2 SD of background noise). In V1 experiments, synapses were considered active when average SB fluorescence was higher than background SB fluorescence plus 1 SD. In mPFC experiments, synapses were segmented using the median filtered GZ channel by a custom algorithm based on image local contrast equalization, image thresholding, and then selected based on their radius. In these mPFC experiments, to avoid any misdetection due to the sporadic presence of GZ expressing somata (local transduction), synapses were accepted for quantitative estimates only when positioned along putative axons (visually identified as short sequences of GZ fluorescing boutons connected by an inter-bouton axonal structure). The normalized SB uptake index was computed at the level of each individual synapse as the ratio of SB uptake and GZ expression, both background subtracted. SB and GZ synaptic fluorescence values were expressed as averages over segmented areas, while the corresponding signal background referred to the surrounding fluorescence (circular region around each segmented synapse). For population comparisons, the index of interest (V1: number of active synapses; mPFC: normalized SB uptake) was averaged among different fields of view each coming from the same subject (V1: 2–5 fields/rat; mPFC: 3 fields/rat). In most experiments acquisition parameters were kept constant. For those experiments where they had to be modified, a linear de-trend was applied to the index of interest (trend estimated on the same experimental group; independent variable: detector gain or detector gain times laser power). All pseudo-color representations of SB fluorescence for the in vivo experiments (color maps) here presented have not been equalized for the sake of comparability. All custom software used for the analysis can be provided upon request.

### Statistical analysis

All statistical analysis was done by routines written in Matlab (Mathworks) and R or using Origin (OriginLab). Student’s *t*-test (paired or unpaired; always two-tailed) was used only after testing for normality by one-sample KS test. Two-sample KS test was used to evaluate differences between distributions of fluorescence data sets. Minimum level of significance was set at 5%. Permutation tests for independent samples (10^4^ permutations; two-tailed) were performed as previously described^55^. B-H correction of *p*-values was applied for multiple comparisons. For both in vitro and in vivo investigations, preliminary experiments were run to evaluate the expected degree and variability of SB uptake and establish the most adequate sample size, with the aim of both reaching a meaningful statistical power and minimizing the number of animals involved in the study. The final set of in vitro and in vivo results showed large SB uptake to noise ratios, indicating that the initial estimates were appropriate (for example, the normalized effect size for V1: 4.0; mPFC acute ketamine: 3.56; power > 0.999). Error bars plot SD or s.e.m. as indicated in the text.

### Data availability

The eGFP-SynaptoZip (GZ) cds sequence used in this study has been deposited at GenBank^®^ with the accession code MF797884 (http://www.ncbi.nlm.nih.gov/Genbank). DNA constructs and data that support the findings of this study are available from the corresponding author upon reasonable request.

## Electronic supplementary material

Supplementary Information

Peer Review File

Description of Additional Supplementary Files

Supplementary Movie 1

Supplementary Movie 2

Supplementary Movie 3
