# Supplementary material for: Functional mapping of brain synapses by the enriching activity-marker SynaptoZip
Source: Nat Commun. 2017 Oct 31;8:1229. doi: 10.1038/s41467-017-01335-4 (PMC5663910; doi:10.1038/s41467-017-01335-4)
Supplement: Supplementary file 1 — Supplementary Information [file 41467_2017_1335_MOESM1_ESM.pdf]

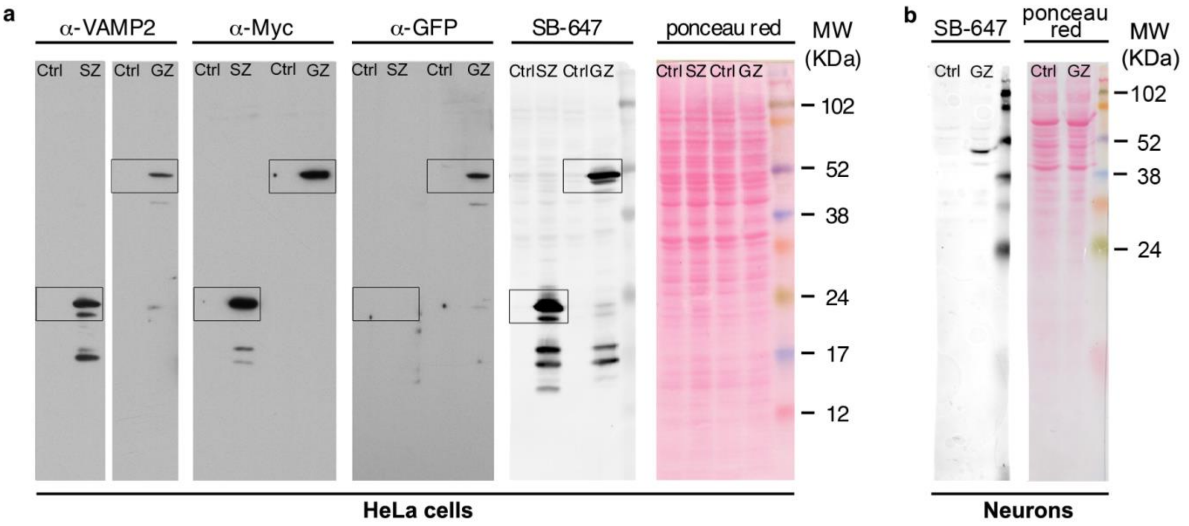

Supplementary Figure 1: WB analysis of transfected cells. a, SDS-PAGE WB of Hela cell lysates from transfected (GZ, SZ) and non-transfected (Ctrl) samples (boxes refers to sections shown in Fig. 1b). Slices of the blotting membrane, containing identical samples, were detected with anti-VAMP2, anti-Myc, anti-GFP antibody, and with SB-Alexa647, as indicated. Different film exposures were used for detection with anti-VAMP2. b, SDS-PAGE WB of cultured CA3-CA1 hippocampal neuron lysates from transduced (GZ) and non-transduced (Ctrl) samples. Rightmost lanes: Ponceau red staining and M.W. markers (same membrane as panel SB-647).

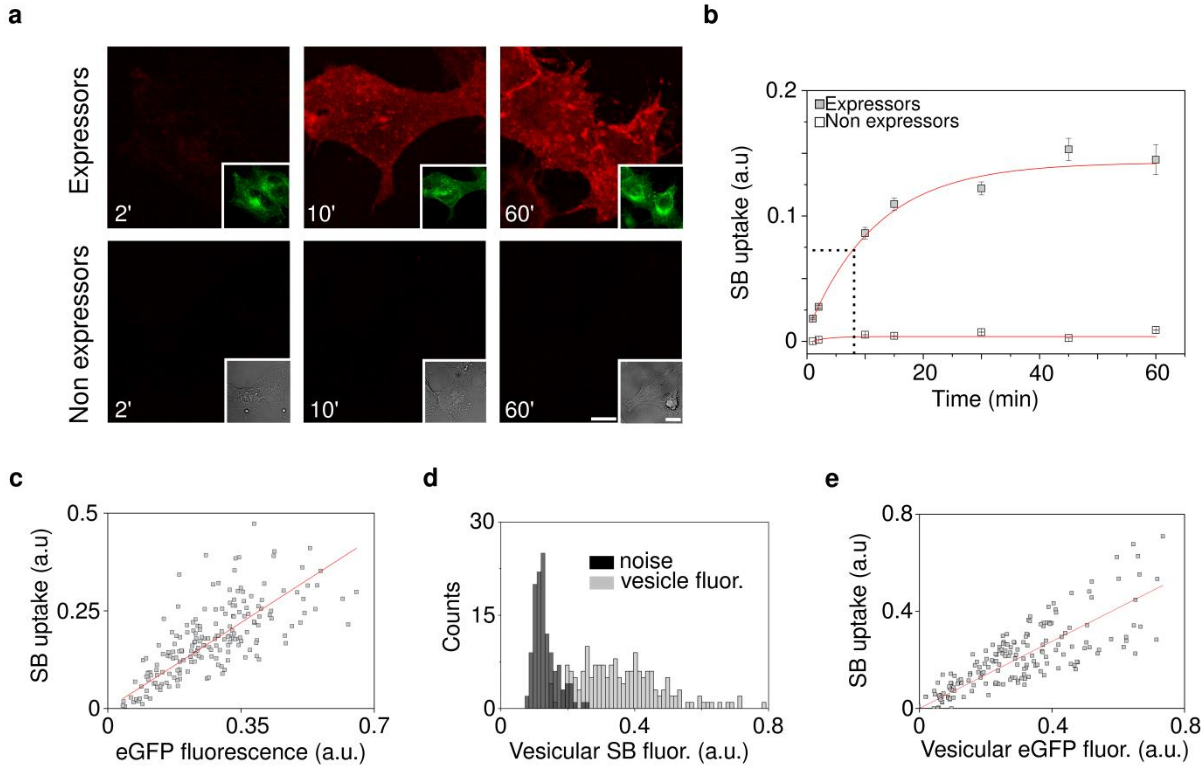

Supplementary Figure 2: Characterization of the SynaptoZip-Synbond reporter in Hela cells and vesicles. a-b, SB uptake by GZ transfected ("expressors") and non-transfected ("non expressors") Hela cells exposed to SB for increasing times (SB 5 nM, 37 °C; 2 – 60 min; N = 19-59 cells per point; always $p < 10^{-4}$; Wilcoxon rank sum test; mean ± SD). a, SB uptake images taken at t = 2, 10 and 60. Insets are downsized pictures showing GZ expression or the DIC image for non-expressors. b, Quantitative analysis of SB uptake from expressors and non-expressors. Notice how at longer time intervals, specific SB uptake approaches a ceiling, presumably due to saturation of cycling binding sites. c, Scatter plots of GZ expression vs SB uptake in individual Hela cells (SB 5 nM, 10 min, 37 °C ; N = 204 cells; adj. R^2 = 0.892, slope = 0.628; $p < 0.001$, Pearson test). d, Distribution of SB uptake inside individual GZ positive vesicles (grey bars; N = 180 vesicles) and near-vesicle background fluorescence (black bars; N = 126 background fields; $p < 0{,}01$, KS test). e, GZ expression vs SB uptake in individual Hela vesicles (N = 180; slope = 0.53, adj. R^2 = 0.879; $p < 0.001$, Pearson test). Scale bar: 10 µm (a).

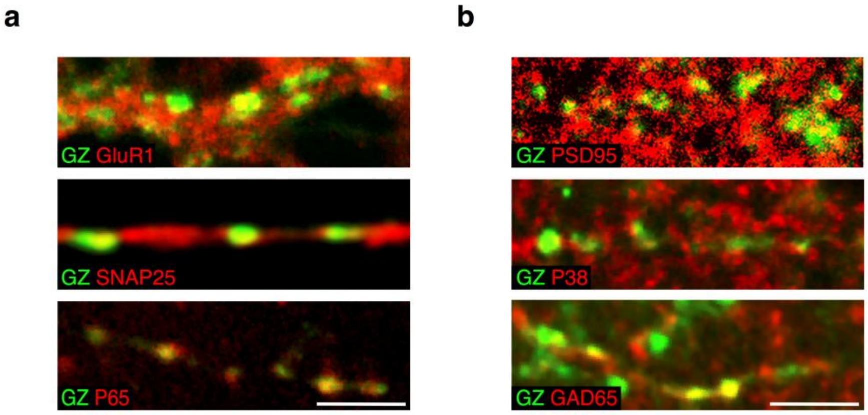

Supplementary Figure 3: Synaptic localization of GZ. a,b, Merged images of GZ expressing synapses (green) in cultured hippocampal neurons (a) and in brain (b; V1 cortex top and middle, mPFC bottom) labeled with antibodies against presynaptic (P38; P65; SNAP25; GAD) and postsynaptic (GluR1; PSD-95) markers (red). Scale bars: 5 µm (a,b).

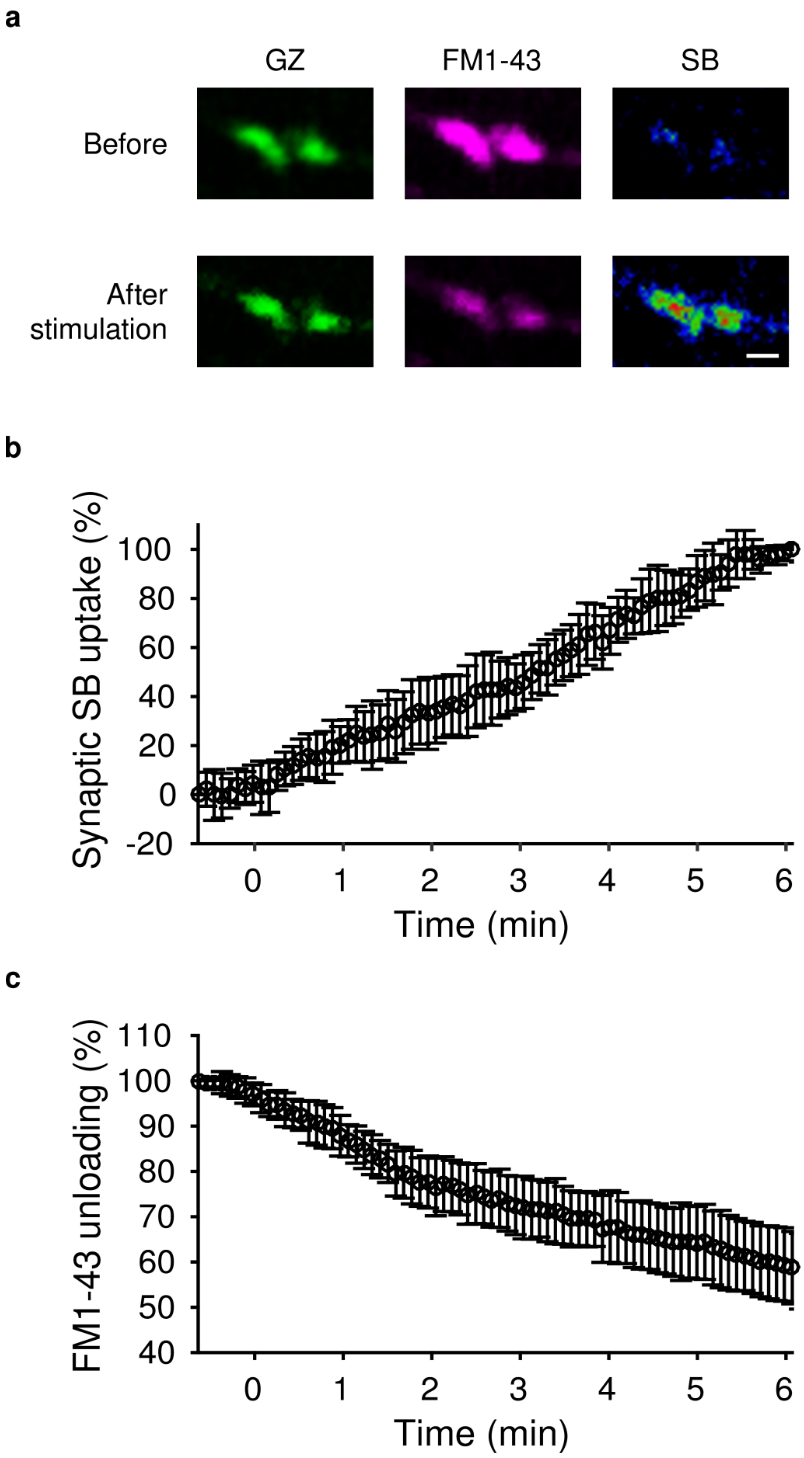

Supplementary Figure 4: SB uptake and simultaneous FM1-43 dye unloading. a, GZ-expressing synapses belonging to cultured CA3-CA1 hippocampal neurons before (top) and 6 min after (bottom) electrical field stimulation (40 mA/cm; 10 APs trains at 10 Hz, 4 s inter-train interval): left, GZ expression (green); middle, FM1-43 dye (purple, dye preloaded with 1-2 min isotonic KCl 90 mM); right, SB (colormap). b, exemplar time-course of SB uptake (normalized for final value) from synapses belonging to the same axon (N = 9). c, time-course of FM1-43 dye unloading (normalized for starting value), same synapses shown in panel (b). Values are mean ± SD. Scale bar: 2 μm (a).

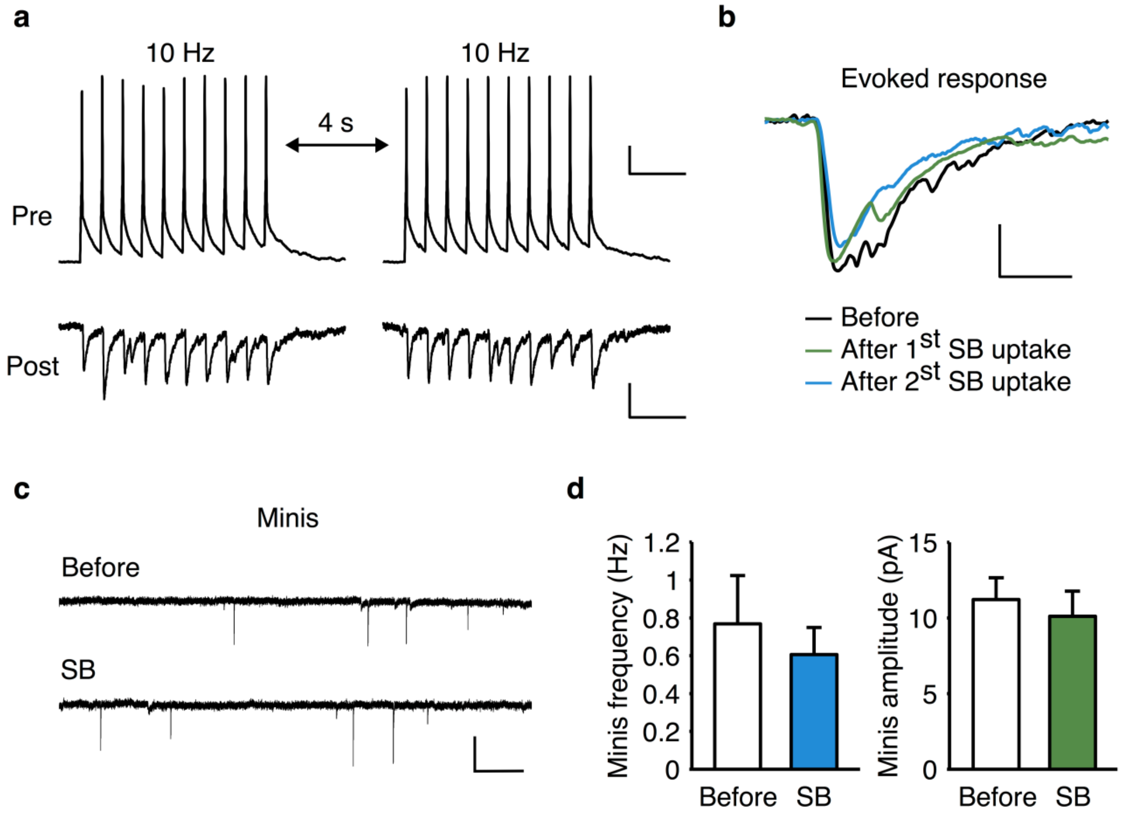

Supplementary Figure 5: Functional synaptic parameters in the presence of SB. a, Exemplar pair-recording to illustrate the stimulation protocol used in Figure 2e-f. A series of trains of action potentials in a GZ expressing cell generates clear postsynaptic responses (10 Hz stimulations, train duration 1 sec, 4 sec inter-train intervals; 5 nM SB present in the bath). b, Superimposition of EPSCs waveforms before and after two epochs of stimulation in the presence of SB (same recording as in a; 5 min stimulation epochs); c,d, Amplitude and frequency of quantal releases in cultured hippocampal neurons before and during the application of extracellular SB (SB 5 nM, 24 °C, TTX 1 μM; miniature frequency before 0.77 ± 0.26 Hz, during SB perfusion 0.60 ± 0.14 Hz, p = 0.31; miniature amplitude before 11.20 ± 1.45 pA, during SB perfusion 10.11 ± 1.65 pA, p = 0.12; N = 5 experiments; mean ± s.e.m.; Wilcoxon two-tailed signed-rank test). Scale bars: 15 mV or 25 pA, 250 ms (a); 50 pA, 20 ms (b).

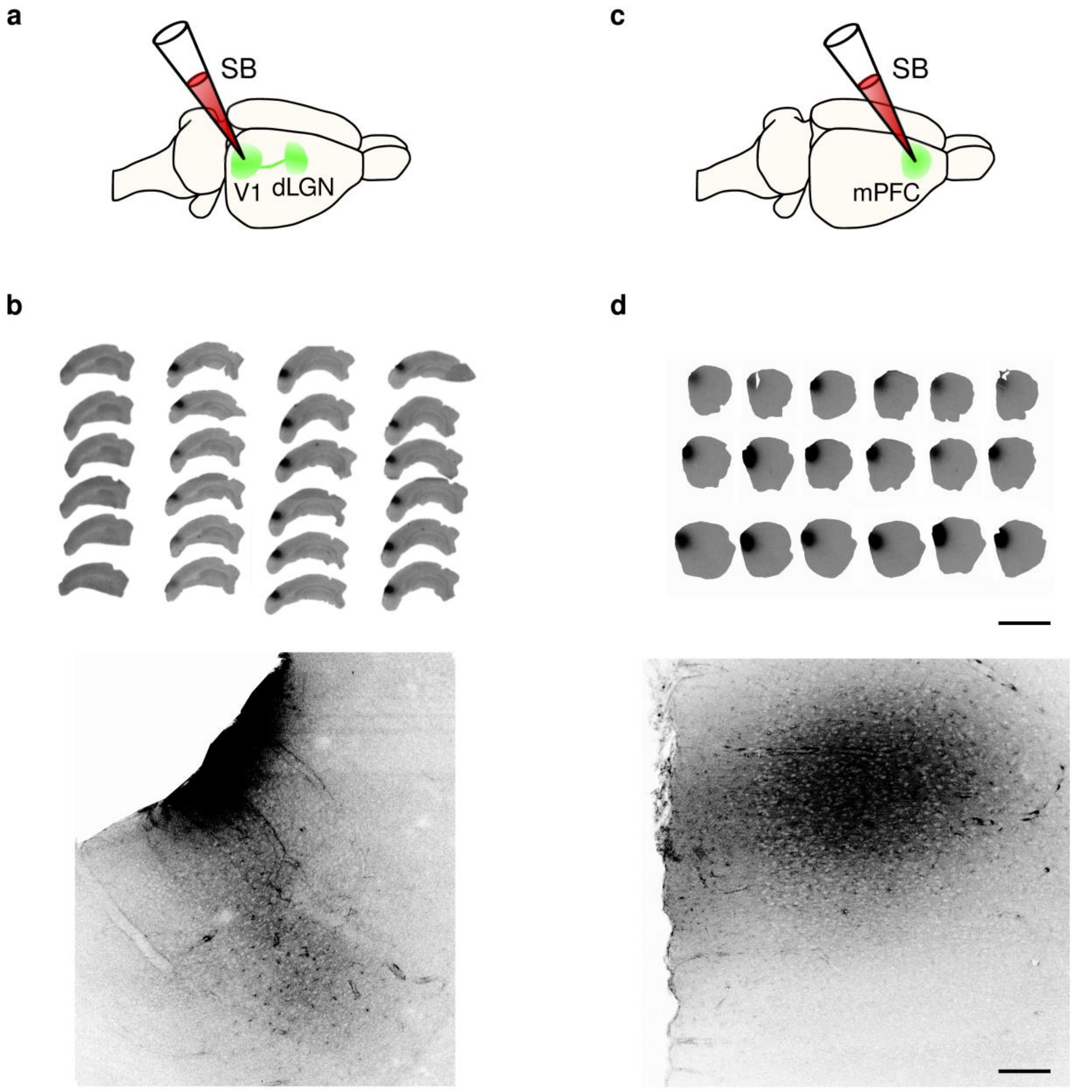

Supplementary Figure 6: *In vivo* delivery of SB into the neocortex. a-d, SB *in vivo* delivery and its accumulation (dark areas) in V1 and mPFC cortices in two representative cases (injection sites: a, superficial V1 layers I-II; b, mPFC, layer V; see methods for details). Cartoons in a,c illustrate the sites of GZ transduction and expression (green) and of SB delivery (red pipette). b,d, SB spread in sequential 40 µm thick coronal slices from representative experiments, magnified slices are shown below. Scale bars: (b,d), top 5 mm, bottom 245 µm.

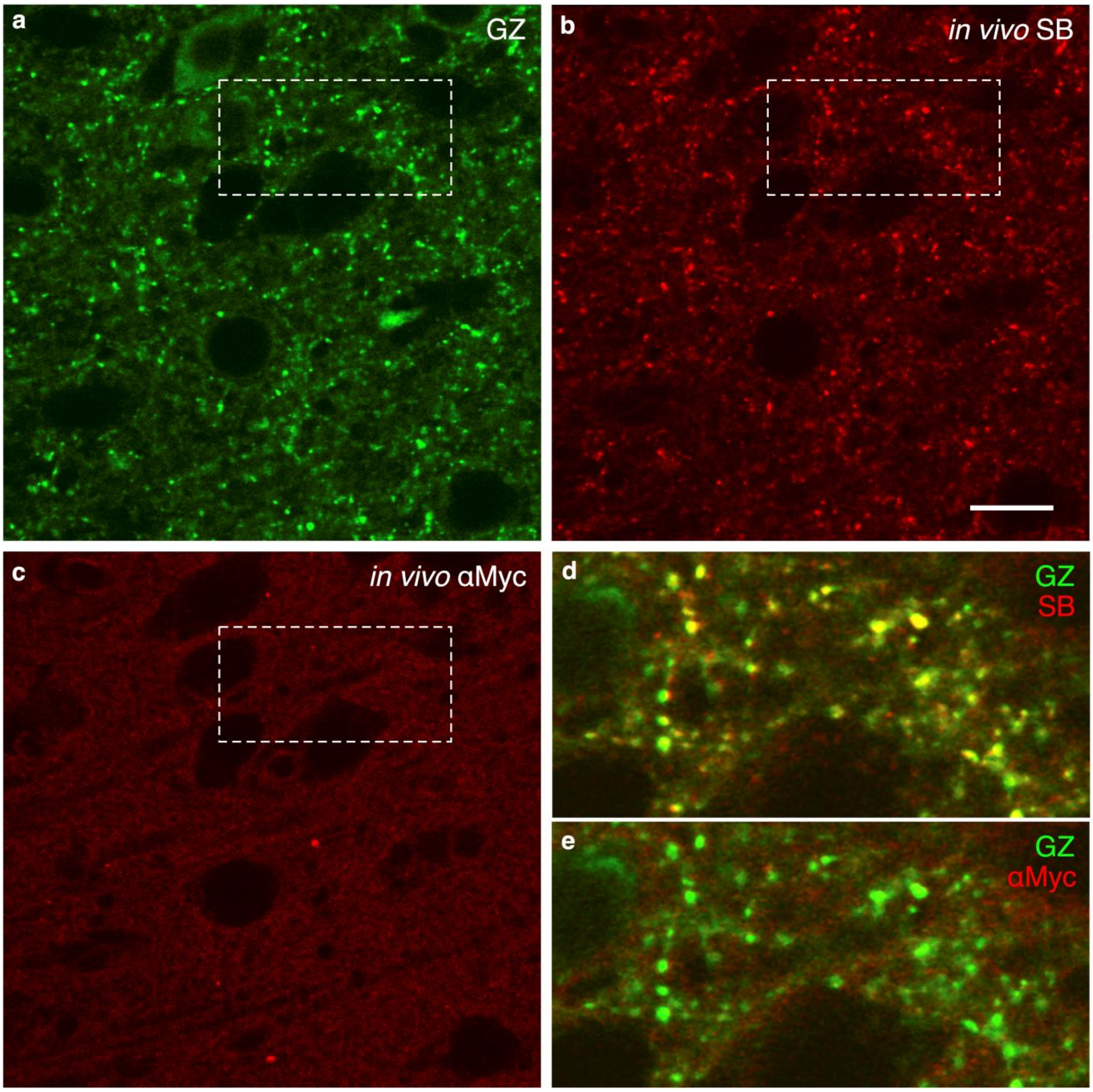

Supplementary Figure 7: SB but not antibodies can access the synapse in the living brain. a-c, GZ expressing mPFC (a) was injected with a mixture of SB-Alexa647 (b) and anti-Myc (c), both directed at the intraluminal portion of GZ and shown here in red. Micro-perfusion protocol as in Figure 5 (injected volume 3 µl; final anti-Myc concentration 0.04-0.2 mg/ml; after fixation and tissue slicing, anti-Myc antibodies were detected by a donkey anti-mouse-Alexa568). d-e, Merged images of GZ with SB (d) or anti-Myc (e). Magnified areas shown in d-e are indicated by dashed boxes in a-c. Notice that the anti-Myc labelling is diffuse and not localized at synaptic sites. On the contrary SB molecules effectively reached mPFC presynaptic boutons and accumulated inside them (yellow color in merged images). Scale bars: (a-c) 10 µm.

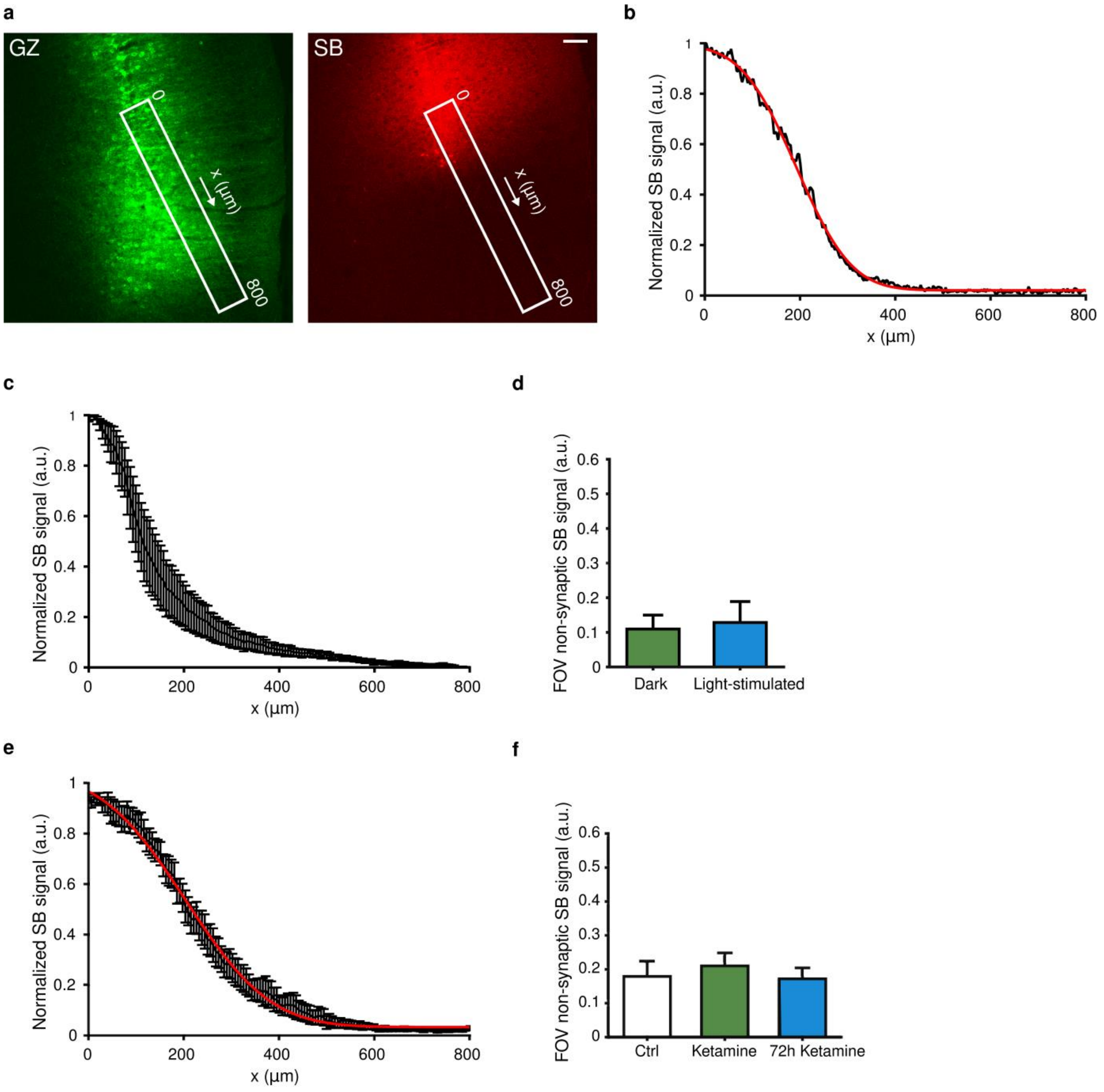

Supplementary Figure 8: Analysis of SB microperfusion in the V1 cortex and mPFC. a, Low magnification images (10x objective) showing GZ (green) and SB (red) fluorescence from an exemplar mPFC experiment (90 min from the end of SB delivery); b, the profile of SB concentration (same mPFC experiment of panel a) from the injection site (x = 0 μm) to the layer of acquisition (x = 600-800 μm). c, pooled SB concentration profiles from V1 microperfusion experiments (N = 5 experiments; 30 min SB microperfusion). d, comparison of non-synaptic SB fluorescence (averaged on the FOV) among the V1 experimental group (N = 5 for each group). e, pooled SB concentration profiles from the mPFC experiments (N = 7 experiments; 90 min from the end of SB delivery). d, comparison of non-synaptic SB fluorescence (averaged on the FOV) in the mPFC experimental groups (N = 6 for each group). In all profile plots, the red line was obtained by fitting with a standard diffusion model. All values are mean ± s.e.m. Scale bar: 100 μm (a).

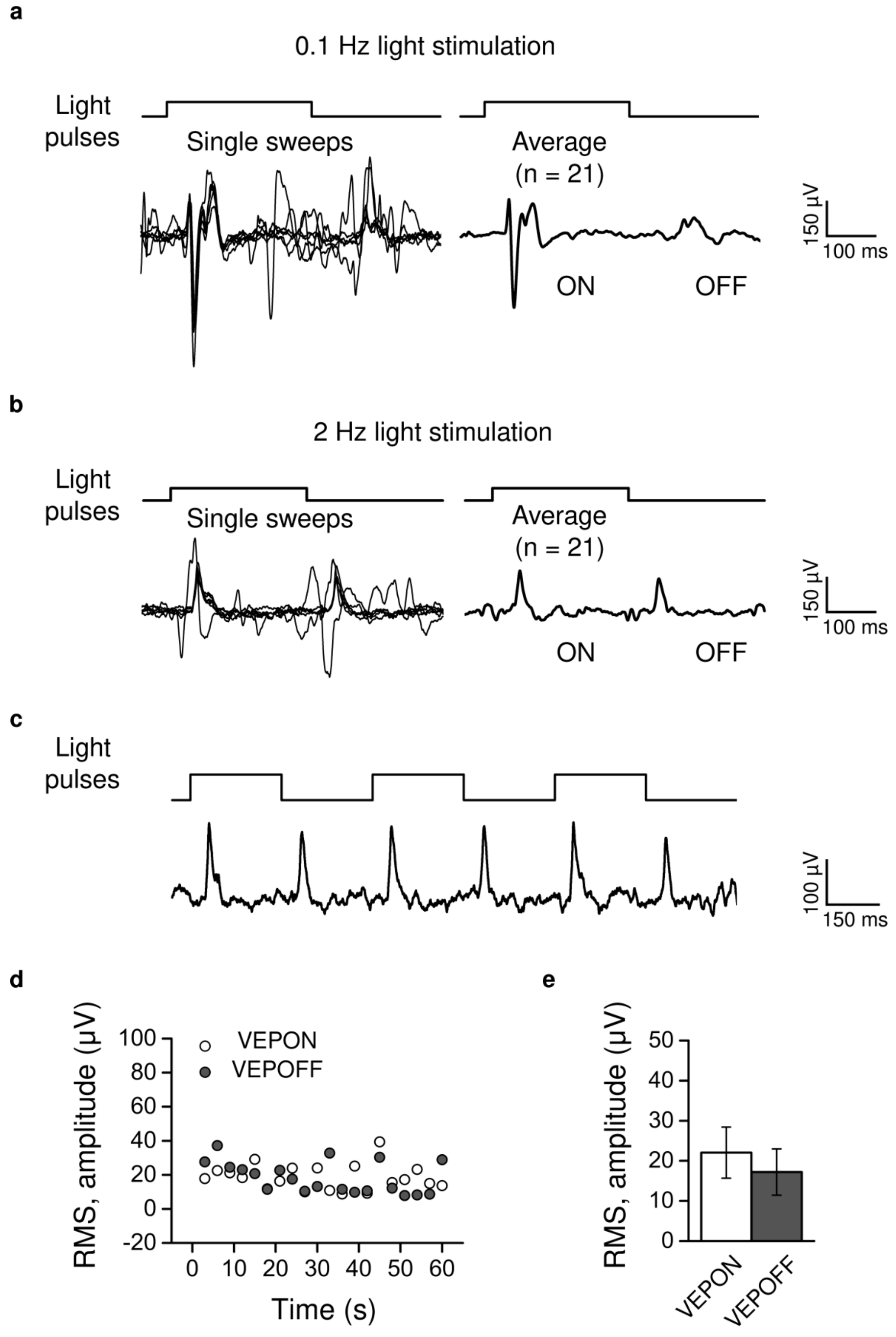

Supplementary Figure 9: Validation of visual stimulation protocol for combined ON and OFF activations of V1 cortex. a, Consecutive visual evoked potentials (VEPs; N = 6 traces; left) evoked with light pulses at 0.1 Hz (pulse duration = 300 ms; pulse irradiance = 150 µW/cm2), and VEP ensemble average (right; N = 21) from one representative experiment (rat V1 cortex; sevoflurane anesthesia; see methods for details). b, consecutive visual evoked potentials (VEPs; N = 6 traces; left) and VEP ensemble average (right; N = 21) evoked in similar conditions as in a but at 2 Hz frequency (the stimulation frequency used for SB uptake experiments; Figure 4). Despite the drop in VEP amplitude, the ON and OFF responses become more alike at higher frequency. c,d, Sequential ON and OFF VEPS in one representative experiment to illustrate their amplitude stability when evoked at 2 Hz. e, RMS amplitude of ON and OFF VEPS evoked at 2 Hz in 60 s trials (N = 5 rats; RMS measured for 125 ms starting from stimulus onset for VEPON, and for 125 ms from the stimulus offset for VEPOFF).

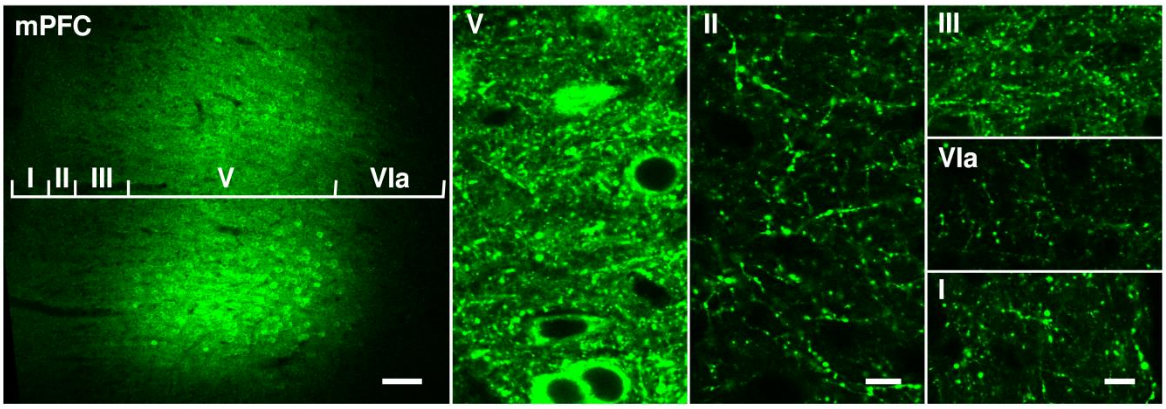

Supplementary Figure 10: GZ expression in different mPFC layers following LV transduction. a, GZ expression in the mPFC 2-3 weeks after transduction (GZ-LV). From the left: low magnification overview with superimposed layer subdivisions; images of each layer at higher magnification, showing brightly fluorescing somata in layer V (the site of transduction) and their synapses across all layers. Presynaptic varicosities along putative axons in layers II and III were those selected for the SB uptake analysis. Scale bars: (a) mPFC, 100 µm; layers V and II, 10 µm; layers III, VIa and I, 10 µm.

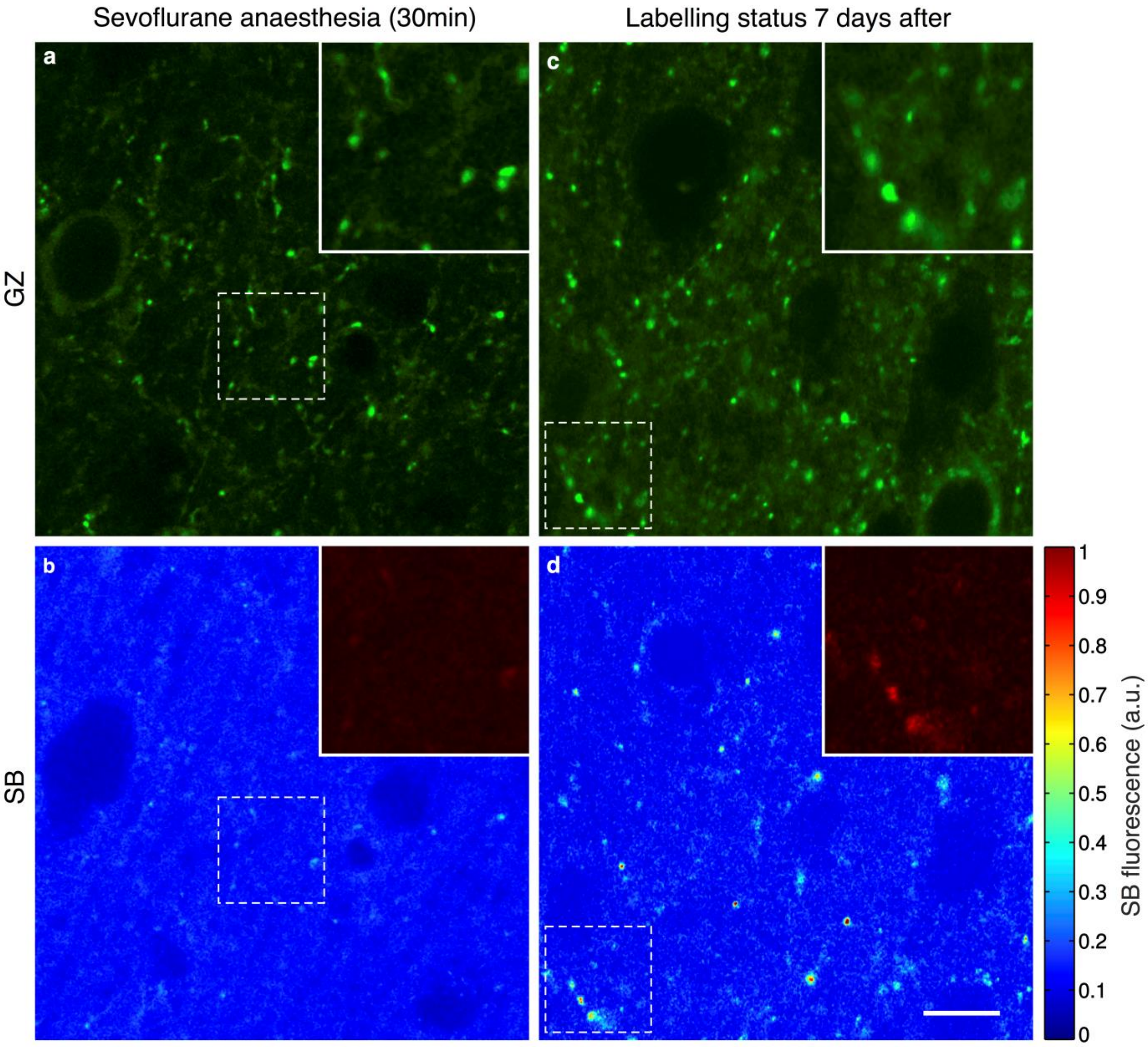

Supplementary Figure 11: Further characterization of SB synaptic labeling in the mPFC. a-b, Very faint SB uptake under sevoflurane anesthesia (30 min, same conditions as in the Sevo epoch of Figure 5b). GZ-expressing synapses in layers II-III (a) and SB signal (b). c-d, Clear evidence for internalized SB at mPFC synapses 7 days after its injection (same conditions as in Figure 5c-d). GZ-expressing synapses in layers II-III (c) and SB signal (d). Insets in b and d are magnified areas with synapses. The color bar is the fluorescence LUT for b and d. Scale bars: (a-d) 10 µm.

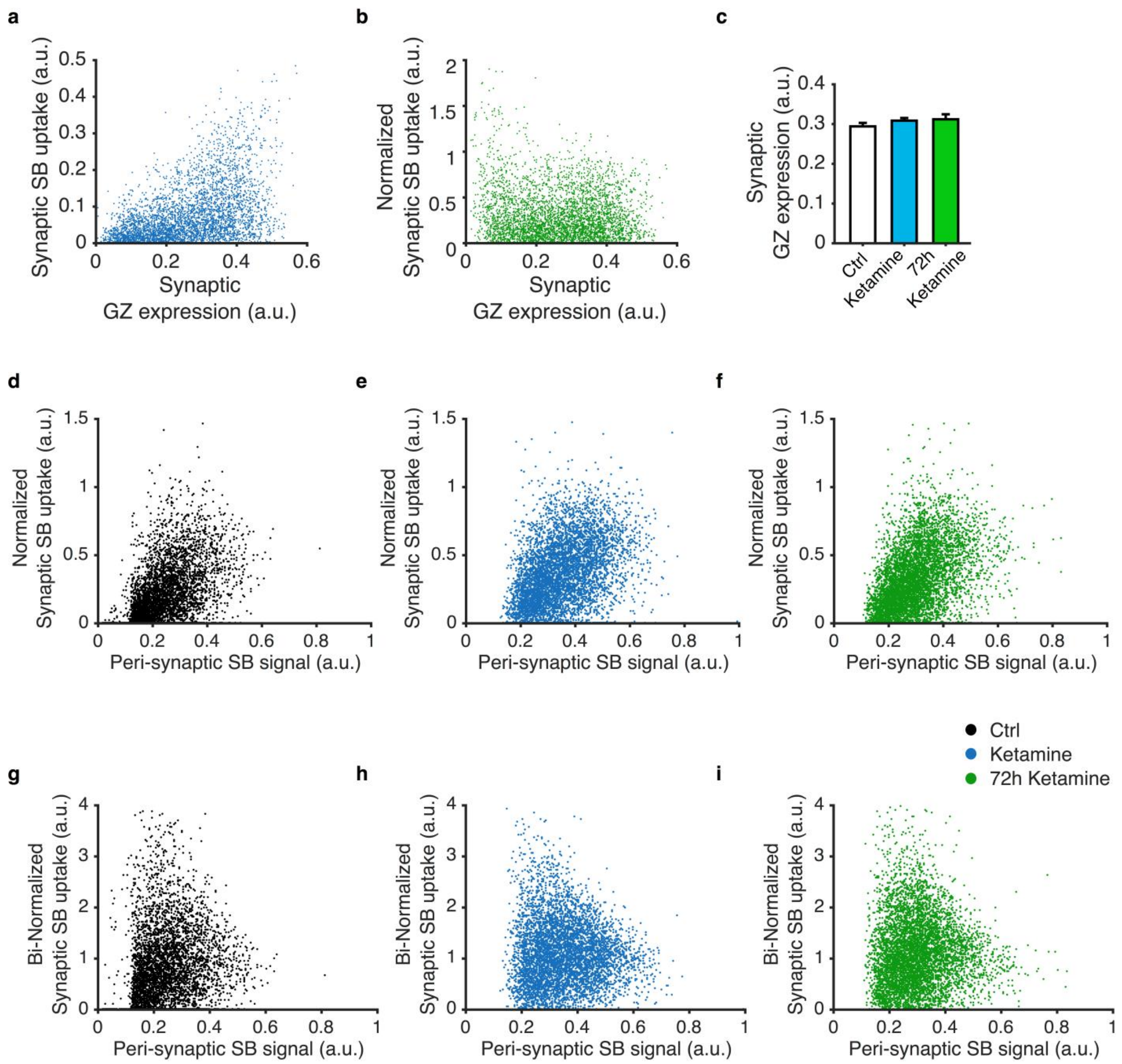

Supplementary Figure 12: Further analysis of SB synaptic labeling in the mPFC. a, Scatter plot of SB uptake vs GZ expression at mPFC synapses (data pooled from the N = 6 control animals, N = 4868 synapses). b, Scatter plot of normalized SB uptake (synaptic SB uptake / GZ expression; same data as in a). c, Bars plot synaptic GZ expression for the three mPFC experimental groups (N = 6 rats for each condition; ctrl, 0.294 ± 0.009; ketamine, 0.309 ± 0.007; 72h after ketamine, 0.312 ± 0.013; mean ± s.e.m; $p_{ctrl\text{-}ket} = 0.68$; $p_{ctrl\text{-}72hket} = 0.68$; $p_{ket\text{-}72hket} = 0.78$; two-samples permutation test, B-H correction). d-f, Synaptic SB uptake (normalized by GZ expression) *versus* peri-synaptic SB fluorescence at individual mPFC synapses from ctrl (d), ketamine (e) and 72h after ketamine (f) groups (plots include all data points used in Figure 5). A significant correlation was detected in all groups (0.4 < ρ < 0.5, p always < 0.0001; Pearson correlation test), indicating that the uptake signal is influenced by the SB concentration found in the extracellular space. g-i, Synaptic SB uptake (as above) divided by peri-synaptic SB fluorescence *versus* the latter at individual mPFC synapses from ctrl (g), ketamine (h) and 72h after ketamine (i) groups (plots include all data points used in Figure 5). The degree of correlation between the uptake and extra-synaptic signal, despite being reduced by the bi-normalization method, remains significant for ctrl (ρ = 0.13, p<0.0001) and 72h after ketamine (ρ = 0.08, p<0.0001) groups (the ketamine

group showed no significant correlation: ρ = 0.02, $p = 0.15$; Pearson correlation test). Therefore the bi-normalization was not used for population comparisons (Figure 5).

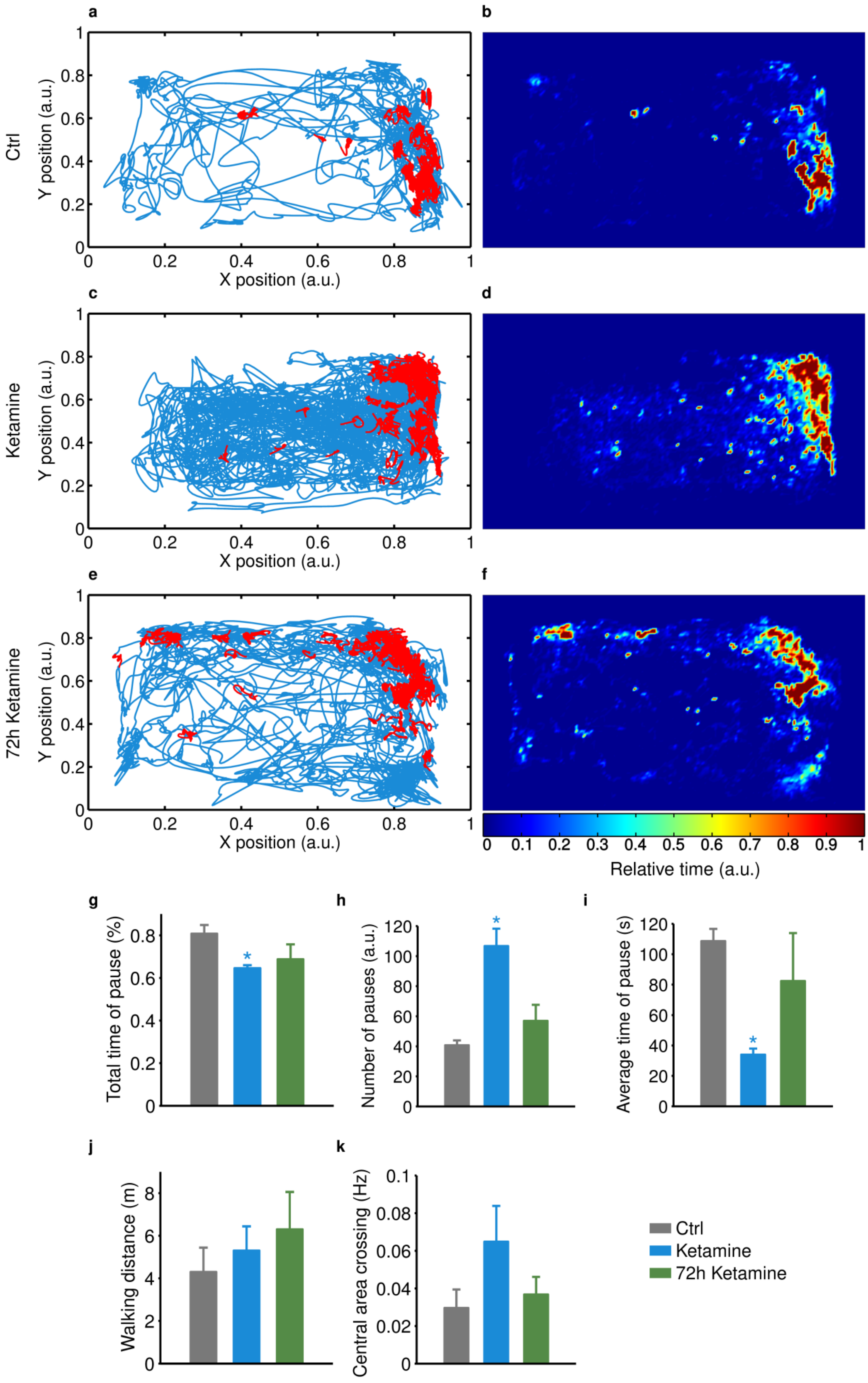

Supplementary Figure 13: Behavioral analysis of ketamine effects at early and late time points.

a-f, Analysis of locomotor behavior in the same rat one day before ketamine treatment (a,b), and after its i.p. injection (c-f; 15 mg/kg), recorded in an early (c,d; starting at 0 h from ketamine injection) and late time

window (e,f; starting at 72 h; observation window 1.5 h). Panels a,c,e depict the rat movement path, red color refers to pauses. Panels b,d,f depict in color scale (LUT at the bottom of panel f) the relative time spent in the different space locations. g-k, Quantitative analysis of locomotor behavior in all conditions in N = 4 rats. g, Total time of pause (ctrl: 0.81 ± 0.04 %; ketamine: 0.65 ± 0.01 %, $p < 0.05$; 72 h after ketamine: 0.69 ± 0.07 %, n.s.); h, number of pauses (ctrl: 41 ± 3; ketamine: 107 ± 11, $p < 0.05$; 72 h after ketamine: 57 ± 11, n.s.); i, average time of pause (ctrl: 109 ± 8 s; ketamine: 34 ± 4 s, $p < 0.005$; 72 h after ketamine: 82 ± 31 s, n.s.); j, walking distance (ctrl: 4.3 ± 1.1 m; ketamine: 5.3 ± 1.1 m, n.s.; 72 h after ketamine: 6.3 ± 1.8, n.s.); k, central area crossing (ctrl: 0.03 ± 0.01 Hz; ketamine: 0.06 ± 0.02 Hz, n.s.; 72 h after ketamine: 0.04 ± 0.01 Hz, n.s.). All values expressed as mean ± s.e.m. Statistical comparisons between treatment and control are paired samples Student's t-tests, p-values are B-H corrected. The water source was positioned in the upper right corner. These experiments were designed to fully match the synaptic SB uptake experiments in the mPFC. Notice how at early times ketamine increases the number of pauses and reduces their average length, a fragmented locomotion indicating ataxia. Overall the total time spent in the same location is significantly reduced by ketamine. The average total walking distance is increased by ketamine at both early and late time points, with a change in the rat exploratory behavior (average increase in the number of central area crossings). Dimension of the observation area: 38 by 32 cm.
