# Supplementary material for: Functional mapping of brain synapses by the enriching activity-marker SynaptoZip
Source: Nat Commun. 2017 Oct 31;8:1229. doi: 10.1038/s41467-017-01335-4 (PMC5663910; doi:10.1038/s41467-017-01335-4)
Supplement: Supplementary file 3 — Description of Additional Supplementary Files [file 41467_2017_1335_MOESM3_ESM.pdf]

File Name: Supplementary Movie 1
Description: **Dynamics of SB labeled vesicles in SZ expressing Hela cells.** Cells were incubated for 30 min in presence of 5 nM SB-Alexa647 (37 °C, 5% CO_2), quickly washed with a Tyrode solution, then placed under the stage of a confocal microscope (63x, N.A. 1.4, oil immersion objective) and imaged alive (24°C). A representative cell is shown in the video (frame interval: 3.569 s). Scale bar: 8 µm.

File Name: Supplementary Movie 2
Description: **Time-lapse imaging of synaptic SB uptake at cultured hippocampal synapses.** The small white square appearing on the top left indicates the occurrence of action potentials firing (trains of 10 APs at 10 Hz, inter-leaved with 4 s pauses; SB 5 nM, 24 °C). Same experiment as in Figure 2e-f (40x, N.A. 0.8, water immersion objective; GZ, green; SB, hot color map; frame interval, 10 s). Scale bar: 4.11 µm.

File Name: Supplementary Movie 3
Description: **3D view of activity-labelling of thalamo-cortical synapses.** Interpolated 3D intensity projection of a confocal Z-stack obtained from layer IV of a light stimulated animal (ON-OFF pulse width 250 ms, intensity 340 µW/cm², at 2 Hz for 15 min; 63x, N.A. 1.4, oil immersion objective). GZ expression on the left (green), SB uptake on the right (red). 2 µm axial resolution, 188 µm x 188 µm field of view.
