# Supplementary material for: Functional mapping of brain synapses by the enriching activity-marker SynaptoZip
Source: Nat Commun. 2017 Oct 31;8:1229. doi: 10.1038/s41467-017-01335-4 (PMC5663910; doi:10.1038/s41467-017-01335-4)
Supplement: Supplementary file 2 — Peer Review File [file 41467_2017_1335_MOESM2_ESM.pdf]

Editorial Note: This manuscript has been previously reviewed at another journal that is not operating a transparent peer review scheme. This document only contains reviewer comments and rebuttal letters for versions considered at *Nature Communications*.

REVIEWERS' COMMENTS:

Reviewer #1 (Remarks to the Author):

The tools for examining the history of activity are still relatively primitive. The enclosed manuscript describes a new approach, which although not without challenges in terms of its use, does represent a significant step forward in the expansion of this tool kit.
The authors show the utility of this approach by examining synaptic activity in-vivo in: 1) visual cortex (Fig 4) where visual stimulation can be mapped at the synapse level; 2) the mPFC, where ketamine strongly impacts synaptic activity in a way that long outlasts the impact on animal motility.

The authors have, in my opinion, answered the queries of all the reviewers adequately and significantly improved both the clarity and the quality of the manuscript.

Reviewer #3:
{{Editor: This referee did not have formal comments to the authors as s/he found the revised paper to be satisfactory and endorses publication.}}
